# Application of Supercritical CO_2_ Extraction and Identification of Polyphenolic Compounds in Three Species of Wild Rose from Kamchatka: *Rosa acicularis*, *Rosa amblyotis*, and *Rosa rugosa*

**DOI:** 10.3390/plants14010059

**Published:** 2024-12-27

**Authors:** Mayya P. Razgonova, Muhammad A. Nawaz, Elena A. Rusakova, Kirill S. Golokhvast

**Affiliations:** 1 N.I. Vavilov All-Russian Institute of Plant Genetic Resources, B. Morskaya 42-44, 190000 Saint-Petersburg, Russia; golokhvast@sfsca.ru; 2 Far Eastern Federal University, Sukhanova 8, 690950 Vladivostok, Russia; 3 Advanced Engineering School “Agrobiotek”, National Research Tomsk State University, Lenin Ave, 36, 634050 Tomsk, Russia; 4 FSBSI Kamchatsky Scientific Research Institute of Agriculture, Centralnaya, 4, 684033 Sosnovka, Russia; rubusarcticus@mail.ru; 5 Siberian Federal Scientific Centre of Agrobiotechnology RAS, Centralnaya 2b, Presidium, 633501 Krasnoobsk, Russia

**Keywords:** *Rosa acicularis*, *Rosa amblyotis*, *Rosa rugosa*, tandem mass spectrometry, SC-CO_2_ extraction, polyphenols, metabolome

## Abstract

A comparative metabolomic study of three varieties of wild Rosa (*Rosa acicularis*, *Rosa amblyotis*, and *Rosa rugosa*) from a Kamchatka expedition (2024) was conducted via extraction with supercritical carbon dioxide modified with ethanol (EtOH), and detection of bioactive compounds was realized via tandem mass spectrometry. Several experimental conditions were investigated in the pressure range 50–350 bar, with the used volume of co-solvent ethanol in the amount of 2% in the liquid phase at a temperature in the range of 31–70 °C. The most effective extraction conditions are the following: pressure 200 Bar and temperature 55 °C for *Rosa acicularis*; pressure 250 Bar and temperature 60 °C for *Rosa amblyotis*; pressure 200 Bar and temperature 60 °C for *Rosa rugosa*. Three varieties of wild *Rosa* contain various phenolic compounds and compounds of other chemical groups with valuable biological activity. Tandem mass spectrometry (HPLC-ESI–ion trap) was applied to detect the target analytes. A total of 283 bioactive compounds (two hundred seventeen compounds from the polyphenol group and sixty-six compounds from other chemical groups) were tentatively identified in extracts from berries of wild *Rosa*. For the first time, forty-eight chemical constituents from the polyphenol group (15 flavones, 14 flavonols, 4 flavan-3-ols, 3 flavanones, 1 phenylpropanoid, 2 gallotannins, 1 ellagitannin, 4 phenolic acids, 1 dihydrochalcone, and 3 coumarins) were identified in supercritical extracts of *R. acicularis*, *R. amblyotis*, and *R. rugosa*.

## 1. Introduction

The genus Rosa (family *Rosaceae*) is represented on the territory of Kamchatka by three species: *Rosa rugosa* Thumb., *Rosa amblyotis* C.A. Mey., and *Rosa acicularis* Lindl. Fresh fruits and leaves contain up to 900 mg% ascorbic acid per dry pulp weight. Fresh petals contain 0.25–0.38% essential oil. Its neutral volatile fraction contains 86.3% phenylethyl alcohol and some linalool, citronellol, geraniol, nerol, etc. Eugenol was found in the phenolic fraction, phenylacetic, benzoic, and other acids in the acid fraction. *R. rugosa* is a medicinal plant widely used in traditional and folk medicine. Extracts of *R. rugosa* have been valued for Asian culinary, cosmetic, and aromatherapy purposes and used in herbal medicines for diabetes mellitus and osteoarthritis [1]. In Korea, *R. rugosa* has been used as a traditional medicine for its anti-inflammatory, antioxidant, hypoglycemic, and hypocholesterolemic properties. These properties may be primarily associated with both the complex of polyphenolic compounds, including tannins, flavonoids, etc., and the terpene chemical group [2]. The medicinal effects seem to be involved in the presence of many phytochemicals in *R. rugosa* extracts, for example flavonoids, phenylpropanoid, tannins, fatty acids, and terpenoids [3].

Phenolic compounds are secondary plant metabolites whose presence and composition are important indicators of the nutraceutical and nutritional quality of fruits, vegetables, and other plants [4,5]. These compounds have an aromatic ring bearing one or more hydroxyl groups, and phenolic structures can range from a simple phenolic molecule to complex high-molecular polymers [6]. These compounds, as one of the most widespread groups of phytochemicals, directly affect plant physiology and morphology. Phenolic compounds can act as phytoalexins [7], pollinator attractants, antifeedants, plant pigmentation factors, and antioxidants and have a protective function against ultraviolet light [8]. A group of polyphenolic compounds directly contributes to the color and flavor characteristics of fruits and vegetables [9]. Natural polyphenolic compounds have been reported to have excellent properties as food preservatives and play a major role in protecting against several pathological disorders in the human body, such as atherosclerosis, brain dysfunction, and cancer (Gordon, 1996). Moreover, polyphenols also have many industrial applications; for example, polyphenols directly obtained from plant extracts can be used as natural colorants and preservatives for food products or in the production of paints, paper, and cosmetics [10].

Supercritical fluid extraction with the use of pressured CO_2_ (SC-CO_2_) has been used since the last 50 years in analytical methodologies to investigate the composition of food products, removal of undesirable substances, and isolation of the polyphenol group of compounds and other chemical constituents. The goal of this work was to identify and select polyphenolic compounds from three varieties of wild *Rosa* (*R. acicularis*, *R. amblyotis*, and *R. rugosa*) via extraction with SC-CO_2_. Also, a tandem mass spectrometry protocol was used for the detailed screening of phytochemicals present in three varieties of wild *Rosa*.

## 2. Materials and Methods

### 2.1. Materials

**Figure 1:**
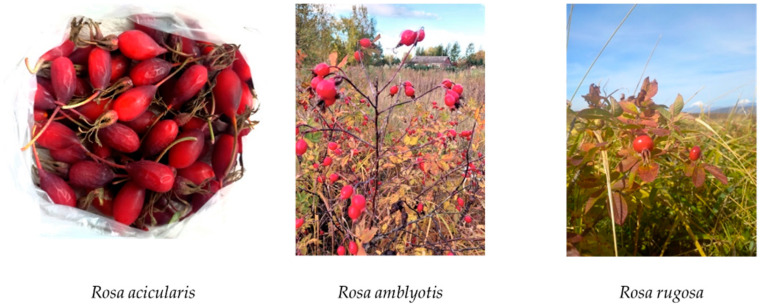
Pictures of the studied three Rosa species.

The subject of this study was the berries of wild *Rosa* (*R. acicularis*, *R. amblyotis*, and *R. rugosa*) collected and grown at the expedition work (Kamchatka) of N.I. Vavilov All-Russian Institute of Plant Genetic Resources, as depicted in Figure 1. The berries of *R. acicularis*, *R. amblyotis*, and *R. rugosa* were harvested at the end of July 2024. All plant tissues used in this work were conformed according to the standard established by the State Pharmacopoeia of the Russian Federation [11].

### 2.2. Chemicals and Reagents

All reagents used in this study were of analytical grade. HPLC-grade acetonitrile was purchased from Fisher Scientific (Southborough, UK), and MS-grade formic acid and ethanol (EtOH) were purchased from Sigma-Aldrich (Steinheim, Germany). Ultrapure water was prepared from Siemens (SIEMENS water technologies, Munich, Germany).

### 2.3. Extraction

SC-CO_2_ extraction was performed using the SFE-500 supercritical pressure extraction system (Thar SCF Waters, Milford, CT, USA). System options include the following: a co-solvent pump (Thar Waters P-50 High Pressure Pump) for the extraction of polar samples; a CO_2_ flow meter (Siemens, Germany) to measure the amount of CO_2_ supplied to the system; and multiple extraction vessels to extract different sample sizes or to increase the throughput of the system. The flow rate was 10–25 mL/min for liquid CO_2_ and 1.00 mL/min for EtOH. Extraction samples of 200 g of berries (*R. acicularis*) were used. Extraction samples of 200 g of berries (*R. amblyotis*) were used. Extraction samples of 200 g of berries (*R. rugosa*) were used. Extraction time was counted after reaching working pressure and equilibrium flow and was 60–90 min for each sample. This method of SC-CO_2_ extraction of plant matrices was tested by the authors on numerous plant samples, including aboveground and underground parts of the plant [12,13].

### 2.4. Liquid Chromatography

HPLC was performed using a Shimadzu LC-20 Prominence HPLC (Shimadzu, Kyoto, Japan) equipped with a UV sensor and C18 silica reverse phase column (4.6 × 150 mm, particle size: 2.7 μm) to perform the separation of multicomponent mixtures. The mobile phase eluent A was deionized water containing 0.1% formic acid, and eluent B was acetonitrile containing 0.1% formic acid. The gradient elution was started at 0–2 min, with 0% eluent B for 2–50 min, and 0–100% B; control washing for 50–60 min with 100% B. The mobile phase flow rate and column temperature were maintained at 0.3 mL/min and 30 °C, respectively. The entire HPLC analysis was performed with a UV–vis detector SPD-20A (Shimadzu, Kyoto, Japan) at a wavelength of 230 nm for the identification of compounds. The injection volume was 10 μL. Additionally, liquid chromatography was combined with a mass spectrometric ion trap to identify compounds.

### 2.5. Mass Spectrometry

MS analysis was performed on an ion trap amaZon SL (Bruker Daltoniks, Leipzig, Germany) equipped with an ESI source in negative ion mode. MS analysis was carried out in electrospray ionization (ESI) mode using negative and positive polarity for all samples with data independent MSE acquisition. The optimized parameters were obtained as follows: ionization source temperature: 70 °C, gas flow: 4 L/min, nebulizer gas (atomizer): 7.3 psi, capillary voltage: 4500 V, end plate bend voltage: 1500 V, fragmentary voltage: 280 V, and collision energy: 60 eV. An ion trap was used in the scan range *m*/*z* 100–1700 for MS and MS/MS. The chemical constituents were identified by comparing their retention index, mass spectra, and mass spectrometry fragmentation with a home-library database built by the Group of Biotechnology, Bioengineering, and Food Systems at the Far-Eastern Federal University (Russia) based on data from other spectroscopic techniques, such as nuclear magnetic resonance, ultraviolet spectroscopy, and MS, as well as data from the literature that is continually updated and revised. The capture rate was one spectrum/s for MS and two spectrum/s for MS/MS. Data collection was controlled by Windows software for Bruker Daltoniks. All experiments were replicated thrice. A four-stage ion separation mode (MS/MS mode) was implemented.

### 2.6. Statistical Analysis

Statistix v 8.1 (https://statistix.informer.com/8.1/ (accessed on 15 February 2021)) was used to compare the global yield extract (mg/100) of the three Rosa varieties according to three-way ANOVA in a completely randomized design. To more clearly present the similarities and differences of bioactive substances identified in different variants of *R. acicularis*, *R. amblyotis*, and *R. rugosa*, the team of authors used the Jaccard index. The Jaccard index, also known as the Jaccard similarity coefficient, is a statistical measure used to evaluate the similarity and diversity of sets of samples. Nine replicate samples were analyzed. A mathematical application, available at https://molbiotools.com/listcompare.php (accessed on 12 September 2024), was used to calculate the Jaccard index in this article. Thee Venn diagram was prepared using an online tool InteractiVenn (https://www.interactivenn.net/ (accessed on 24 August 2023)).

## 3. Results

### 3.1. SC-CO_2_ Extraction of Berries (R. acicularis, R. amblyotis, and R. rugosa)

Three *Rosa* varieties, i.e., *R. acicularis*, *R. amblyotis*, and *R. rugosa*, were examined by SC-CO_2_ extraction under different extraction conditions. The supercritical pressures applied ranged from 100 to 400 bar, and the extraction temperature ranged from 31 to 70 °C. The co-solvent EtOH was used in an amount of 2% of the total solvent amount. Table 1 shows the global yield of bioactive compounds by SC-CO_2_ extraction in three Rosa species. Overall, we observed that temperature and pressure in different Rosa varieties can affect the global yield.

The maximum global yield of bioactive substances from berries of *R. acicularis* was observed under the following extraction conditions:

- With a pressure of 200 Bar, extraction temperature of 55 °C, and extraction time of 1 h, the global yield of biologically active substances was 6.2 mg/100 mg of plant sample; the share of the EtOH modifier was 2%.

In the case of *R. amblyotis*, it was observed that the maximum global yield of bioactive substances was observed under the following extraction conditions:

- With a pressure of 250 Bar, extraction temperature of 60 °C, and extraction time of 1 h, the global yield of biologically active substances was 6.4 mg/100 mg of plant sample; the share of the EtOH modifier was 2%.

It was observed that the maximum global yield of bioactive substances from berries (*R. rugosa*) was observed under the following extraction conditions:

- With a pressure of 200 Bar, extraction temperature of 60 °C, and extraction time of 1 h, the global yield of biologically active substances was 6.7 mg/100 mg of plant sample; the share of the EtOH modifier was 2%.

### 3.2. Global Metabolome Profile of R. acicularis, R. amblyotis, and R. rugosa

**Figure 2:**
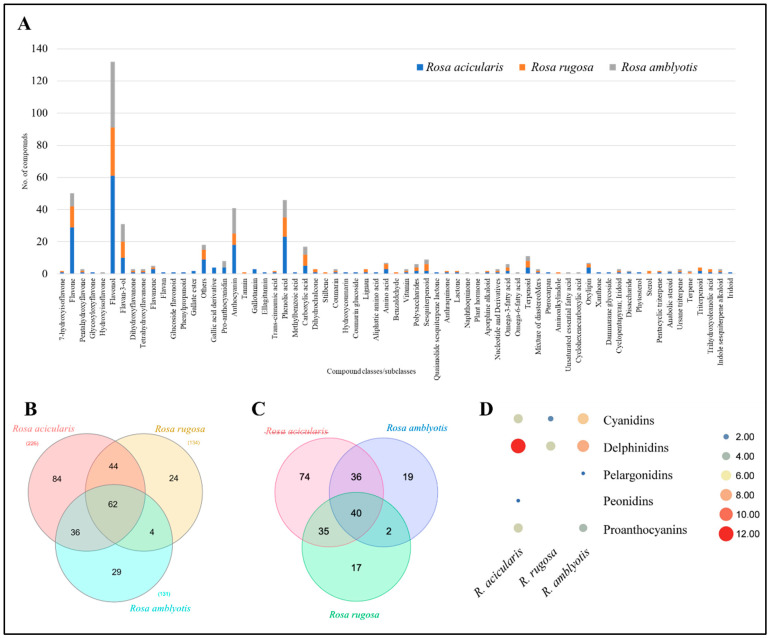
Global metabolome profile of three Rosa species. (**A**) Bar plot showing compound classes (and subclasses) and respective number of compounds detected in each Rosa species. (**B**) Venn diagram showing number of common and specific compounds detected in three Rosa species. (**C**) Venn diagram showing number of common and specific polyphenolic compounds detected in three Rosa species. (**D**) Scatter plot showing number of anthocyanins detected in each Rosa species.

The structural identification of each compound was carried out on the basis of its accurate mass and MS/MS fragmentation by HPLC-ESI-ion trap-MS/MS. We were able to identify 283 chemical compounds from the extracts of *R. acicularis*, *R. amblyotis*, and *R. rugosa*: 217 chemical compounds from the polyphenol group and 66 chemical compounds from other chemical groups. The other groups included amino acids, benzaldehydes, carboxylic acids, terpenoids (sesquiterpenoids and triterpenoids), sterols, iridoids, oxylipins, fatty acids, etc. (Figure 2A). Of the 283 compounds, 62 were commonly detected from all three Rosa species. Of the three species, *R. acicularis* was the richest in the diversity of the detected compounds with 84 uniquely detected compounds followed by *R. amblyotis* (29) and *R. rugosa* (24) (Figure 2B). All identified polyphenols and other compounds along with molecular formulae and MS/MS data for *R. acicularis*, *R. amblyotis*, and *R. rugosa* are summarized in Appendix A, Table A1. The polyphenols detected in our study were categorized as flavones, flavonols, flavan-3-ols, anthocyanidins, phenolic acids, chalcones, lignans, stilbenes, coumarins, etc. In total, the metabolites detected in our study belonged to 18 compound classes. The highest number of metabolites were flavonols (71), followed by flavones (38), flavan-3-ols (13), proanthocyanidins (5), anthocyanins (24), and phenolic acids (32). These numbers indicate that SC-CO_2_ extracts of *R. acicularis*, *R. amblyotis*, and *R. rugosa* are rich in flavonoids. The highest number of chemical compounds from other groups are oxylipins (6) and terpenoids (7).

A Venn diagram showing the similarities and differences in the presence of various polyphenols in *Rosa* varieties (*R. acicularis*, *R. amblyotis*, and *R. rugosa*) is shown in Figure 2C. Forty compounds were commonly detected from the three *Rosa* varieties. These forty compounds belong to compound classes such as flavones, flavonols, anthocyanins, phenolic acids, etc., as major active compounds in SC-CO_2_ extracts of *R. acicularis*, *R. amblyotis*, and *R. rugosa*. The applied methods were able to detect 188 (*R. acicularis*), 97 (*R. amblyotis*), and 95 (*R. rugosa*) polyphenolic compounds.

Moreover, to present the similarities and differences in bioactive substances in different variations of Rosa, we used the Jaccard index (Table 2). The Jaccard index, also known as the Jaccard similarity coefficient, is a statistic used to evaluate the similarity and diversity of sets of samples [14,15]. It showed that the highest degree of similarity is present between the varieties *R. acicularis* and *R. amblyotis*—0.3689.

Twenty-nine compounds classified as anthocyanins (24) and pro-anthocyanins (five) were detected from the studied Rosa species. Generally, *R. acicularis* was rich in anthocyanins; except pelargonidins, other classes were detected. Next, *R. amblyotis* also exhibited cyanidins, delphinidins, and pelargonidins but not peonidin. *Rosa rugosa* had only seven anthocyanins belonging to cyanidins, delphinidins, and peonidin. However, it was deficient in pro-anthocyanidins (Figure 2D; Appendix A, Table A1).

To present the similarities and differences in bioactive substances in different variations of Rosa, we used the Jaccard index (Table 3). It showed that the highest degree of similarity is present between the varieties *R. acicularis* and *R. amblyotis*—0.4483.

#### 3.2.1. Hydroxy(iso)flavones

**Figure 3:**
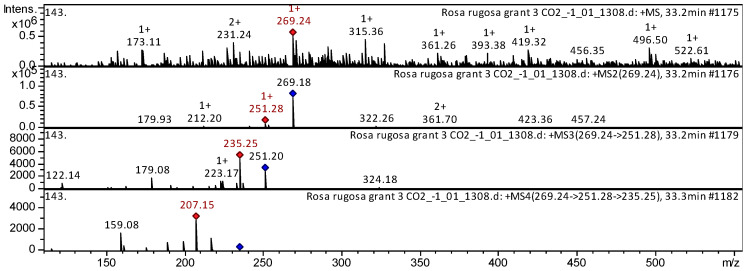
CID spectrum of formononetin from SC-CO_2_ extracts of *R. rugosa*, *m*/*z* 269.24. Above is an MS scan in the range 100–1700 *m*/*z* and below is fragmentation spectra (top to bottom): MS2 of protonated formononetin ion (269.24 *m*/*z*, red diamond), MS3 fragment 269.24 → 251.28 *m*/*z*, and MS4 fragment 269.24 → 251.28 → 235.25 *m*/*z*.

The 7-hydroxyisoflavone formononetin (compound **1**) has been already characterized as a component of *Dracocephalum jacutense* [16], *Medicago varia* [17], *Maackia amurense* [18], and Chinese herbal formula Jian-Pi-Yi-Shen pill [19]. Thus, our results indicate that the flavone formononetin is tentatively identified in the SC-CO_2_ extracts of both *R. acicularis* and *R. rugosa* (Appendix A, Table A1). The CID spectrum (collision-induced spectrum) in the positive ion mode for the flavone formononetin from the *R. rugosa* extract is shown in Figure 3. [M + H]^+^ ion produced three fragment ions with *m*/*z* 251.28, *m*/*z* 212.20, and *m*/*z* 179.93 (Figure 3). The fragment ion with *m*/*z* 251.28 produced three characteristic daughter ions with *m*/*z* 235.25, *m*/*z* 223.17, and *m*/*z* 179.08.

#### 3.2.2. Dihydroxyflavones

**Figure 4:**
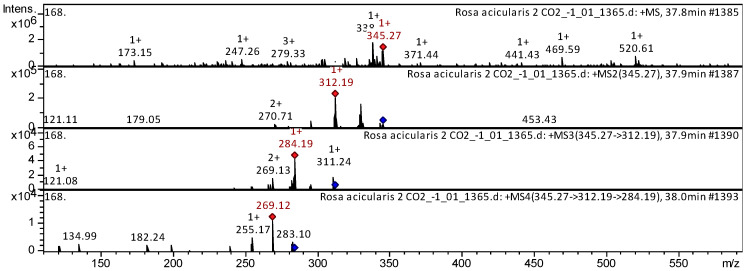
CID spectrum of nevadensin from SC-CO_2_ extracts of *R. acicularis*, *m*/*z* 345.27. Above is an MS scan in the range 100–1700 *m*/*z* and below is fragmentation spectra (top to bottom): MS2 of protonated nevadensin ion (345.27 *m*/*z*, red diamond), MS3 fragment 345.27 → 312.19 *m*/*z*, and MS4 fragment 345.27 → 312.19 → 284.19 *m*/*z*.

The flavones acacetin (compound **3**), cirsimaritin (compound **10**), cirsilineol (compound **17**), dihydroxy-dimethoxy(iso)flavone (compound **11**), and nevadensin (compound **12**) (Appendix A, Table A1) have been already characterized as a component of *Triticum aestivum* [20], *Mentha* [21], *Mexican lupine* species [22], *Artemisia annua* [23], *Rosmarinus officinalis* [24], and *Medicago varia* [17]. We also tentatively identified these flavones in SC-CO_2_ extracts of *R. acicularis*, *R. amblyotis*, and *R. rugosa*. The CID spectrum in positive ion modes for nevadensin from *R. acicularis* extracts is shown in Figure 4. The [M + H]^+^ ion produced three fragment ions with *m*/*z* 312.19, *m*/*z* 270.71, and *m*/*z* 179.05 (Figure 4). The fragment ion with *m*/*z* 312.19 produced two characteristic daughter ions with *m*/*z* 284.19 and *m*/*z* 269.13.

#### 3.2.3. Trihydroxyflavones

**Figure 5:**
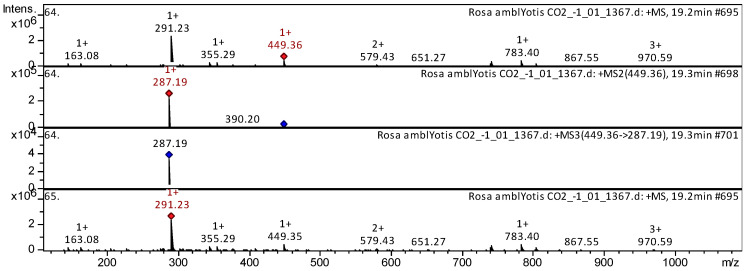
CID spectrum of luteolin-7-*O*-glucoside from SC-CO_2_ extracts of *R. amblyotis*, *m*/*z* 449.36. Above is an MS scan in the range 100–1700 *m*/*z* and below is fragmentation spectra (top to bottom): MS2 of protonated luteolin-7-*O*-glucoside ion (449.36 *m*/*z*, red diamond), MS3 fragment 449.36 → 287.19 *m*/*z*, and MS4 fragment 449.36 → 287.19 → 287.19 *m*/*z*.

The flavones apigenin (compound **2**), jaceosidin (compound **14**), 5,6,4′-trihydroxy-7,8-dimethoxyflavone (compound **16**), cirsiliol (compound **17**), chrysoeriol [chrysoeriol] (compound **8**), luteolin 7-*O*-glucoside (compound **25**), kaempferide (compound **41**), etc. (Appendix A, Table A1), have already been characterized as a component of *Inula gaveolens* [25], *Lonicera henryi* [26], *Ribes meyeri* [27], and *Andean blueberry* [28]. Trihydroxyflavones were tentatively identified in SC-CO_2_ extracts of *R. acicularis*, *R. amblyotis*, and *R. rugosa*. The CID spectrum in positive ion modes for luteolin 7-*O*-glucoside from SC-CO_2_ extracts of *R. amblyotis* is shown in Figure 5. The [M + H]^+^ ion produced one fragment ion with *m*/*z* 287.19 (Figure 5). The luteolin 7-*O*-glucoside was identified in the bibliography in extracts from *Vaccinium macrocarpon* [29] and *Lonicera henryi* [26].

#### 3.2.4. Anthocyanins

**Figure 6:**
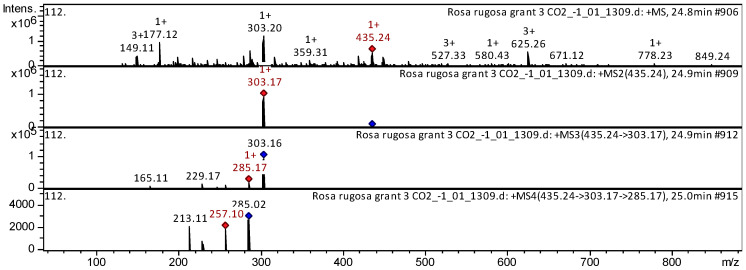
CID spectrum of delphinidin *O*-pentoside from SC-CO_2_ extracts of *R. rugosas*, *m*/*z* 435.24. Above is an MS scan in the range 100–1700 *m*/*z* and below is fragmentation spectra (top to bottom): MS2 of protonated delphinidin *O*-pentoside ion (435.24 *m*/*z*, red diamond), MS3 fragment 435.24 → 303.17 *m*/*z*, and MS4 fragment 435.24 → 303.17 → 285.17 *m*/*z*.

Delphinidin *O*-pentoside (compound **149**), cyanidin-3-*O*-glucoside (compound **151**), delphinidin 3-*O*-glucoside (compound **154**), delphinidin 3-*O*-hexuronide (compound **157**), cyanidin 3-*O*-coumaroyl hexoside (compound **161**), etc. (Appendix A, Table A1), have already been characterized as a component of *Ribes magellanicum*; *Berberis ilicifolia*; *Berberis empetrifolia*; *Ribes maellanicum*; *Ribes cucullatum*; and *Myrteola nummalaria*. Their anthocyanins were tentatively identified in SC-CO_2_ extracts of *R. acicularis*, *R. amblyotis*, and *R. rugosa*. The CID spectrum in positive ion modes for delphinidin *O*-pentoside from SC-CO_2_ extracts of *R. rugosa* is shown in Figure 6. The [M + H]^+^ ion produced one fragment ion with *m*/*z* 303.17 (Figure 6). The fragment ion with *m*/*z* 303.17 produced three characteristic daughter ions with *m*/*z* 285.17, *m*/*z* 229.17, and *m*/*z* 165.11. The anthocyanin delphinidin *O*-pentoside was tentatively identified in the bibliography in extracts from *Myrtle* [30], *Gaultheria mucronate*, *Gaultheria antarctica* [31], and *Andean blueberry* [28].

### 3.3. Newly Detected Chemical Compounds in SC-CO_2_ Extracts of R. acicularis, R. amblyotis, and R. rugosa

Of the detected metabolites in SC-CO_2_ extracts of *R. acicularis*, *R. amblyotis*, and *R. rugosa*, forty-eight chemical constituents from the polyphenol group (15 flavones, 14 flavonols, 4 flavan-3-ols, 3 flavanones, 1 phenylpropanoid, 2 gallotannins, 1 ellagitannin, 4 phenolic acids, 1 dihydrochalcone, and 3 coumarins) were identified for the first time. The newly identified polyphenols include flavones (acacetin, calycosin, diosmetin, odoratin, salvigenin, jaceosidin, nevadensin, cirsilineol, scutellarin, chrysoeriol 6-O-hexoside, chrysoeriol 6-*O*-glucoside, chrysoeriol 8-*O*-glucoside, chrysin 6-*C*-[2″-*O*-glucoside] -glucoside, and chrysin di-*O*-glucoside, lonicerin), flavonols (rhamnetin I, rhamnetin II, taxifolin 3-*O*-arabinofuranoside, isorhamnetin-3-*O*-arabinoside, isorhamnetin 3-*O*-pentoside, astragalin, dihydrokaempferol-O-hexoside, aromadendrin O-hexoside, taxifolin-3-*O*-glucoside, taxifolin-3-*O*-hexoside, quercetin 7-*O*-glucoside, quercetin-hexuronide, quercetin *O*-hexoside *O*-deoxyhexoside, and bioquercetin), flavan-3-ols (afzelechin, (*epi*)-afzelechin, gallocatechin, and (*epi*)-gallocatechin), flavanones (eriodictyol, hesperitin, and taxifolin-3-*O*-rhamnoside), phenylpropanoid vimalin, etc. (Appendix A, Table A1).

## 4. Discussion

Since the most biologically active substances in plants are usually polyphenolic compounds, extraction is the first and most important step in the stepwise separation of these compounds [32]. The most common extractions applicable to phenolic compounds are liquid/liquid and solid/liquid, and they are often used specifically for the separation of these components. The phenolic structure of polyphenols makes them relatively hydrophilic, so free phenolic compounds, including aglycones, glycosides, and oligomers are extracted using water, polar organic solvents such as ethyl acetate, methanol, ethanol, acetonitrile, acetone, and their mixtures with water or non-polar solvents (chloroform and diethyl ether) [4]. Nowadays, as a result of the negative impact of industrial extraction on the environment, the concept of green extraction has been introduced to protect both the environment and consumers. This also directly affects the increasing competition of industries to be more environmentally friendly (use of by-products and biodegradability), economical (lower energy and solvent consumption), and innovative [33]. In line with this approach to green extraction, non-traditional extraction methods such as microwave, ultrasonic extraction, and methods based on the use of compressed fluids as extracting agents such as subcritical water extraction (SWE), supercritical fluid extraction (SFE), pressurized fluid extraction (PFE), or accelerated solvent extraction (ASE) are used for the actual separation of phenolic compounds [34,35]. Generally, the most common extraction methods for phenolic compounds use acetone [36,37] or methanol [38] as phenolic compound extracting agents, but from the point of view of use in food and cosmetic industries, ethanol and water are definitely preferred [39].

As shown earlier, bioactive compounds of aboveground plant parts are efficiently extracted using organic solvents such as methanol and ethanol. However, the extraction products at the final stage require additional costs in terms of purification from trace amounts of the solvents used. SC-CO_2_ extraction, which is rightfully classified as a “green” extraction method, can be used as an alternative to traditional extraction methods: maceration or Soxhlet extraction [40,41]. SC-CO_2_ extraction has been used in the evaluation of food products, the isolation of bioactive substances, and the determination of lipid levels in food products and the levels of toxic substances. With SC-CO_2_ extraction, the products do not contain residues of organic solvents that are encountered in conventional extraction methods, and the solvents can be toxic, for example in the case of methanol and n-hexane. Easy removal of solvent from the final product, high selectivity, and moderate extraction temperatures are the main attractive features of SC-CO_2_ technology, which leads to a significant increase in research for applications in cosmetic, pharmaceutical, and food industries. When comparing possible supercritical solvents, carbon dioxide has the most attractive advantages: non-toxic, non-flammable, environmentally friendly, and, most importantly, a renewable resource [42]. Previously, our group of authors successfully used supercritical CO_2_ extraction in the extraction of the polyphenolic composition of the forage grass *Medicago varia* [17], the extraction of the aromatic component from *Ribes fragrans* leaves [43], the refinement of the polyphenolic composition of medicinal plants *Ledum palustre* [44] and *Maackia amurense* [18], the isolation of the sulfate compounds from *Zostera marina* [45], etc. Thus, the use of SC-CO_2_ extraction is an effective approach for the extraction of bioactive compounds.

High oxidation–reduction potential is the most important property of flavonoids, which allows them to act as singlet hydrogen quenchers, reducing agents, and hydrogen donors. Flavonoids have metal chelating potential [46]. Regular consumption of flavonoids has been directly associated with a reduced incidence of various cancers and heart disease [47]. There is currently great interest in flavonoid research due to the possibility of direct effects on health through diet, in which case preventive health care can be achieved through the consumption of fruits and vegetables. Flavonols are a class of flavonoids commonly found in many fruits and vegetables, and their content varies greatly depending on environmental factors such as growing conditions, climate, storage, and cooking conditions [48].

A total of 75 flavonols have been identified in SC-CO_2_ extracts of *R. acicularis*, *R. amblyotis*, and *R. rugosa*, of which 14 compounds (rhamnetin I, rhamnetin II, taxifolin 3-*O*-arabinofuranoside, isorhamnetin-3-*O*-arabinoside, isorhamnetin 3-*O*-pentoside, astragalin, dihydrokaempferol-*O*-hexoside, aromadendrin *O*-hexoside, taxifolin-3-*O*-glucoside, taxifolin-3-*O*-hexoside, quercetin 7-*O*-glucoside, quercetin-hexuronide, quercetin *O*-hexoside *O*-deoxyhexoside, and bioquercetin) were identified for the first time in *Rose* SC-CO_2_ extracts, highlighting the great potential for applications in functional food technologies.

Flavanones can be characterized by the presence of a saturated three-carbon chain and an oxygen atom at the C4 position. They are usually glycosylated with a disaccharide at the C7 position. Flavanones are present in high concentrations only in citrus fruits but have also been identified in tomatoes and some aromatic plants (mint). The main aglycones are naringenin in grapefruit, hesperetin in oranges, and eriodictyol in lemons. A total of five flavanones have been identified in berry extracts, of which three compounds (hesperetin, quercetin *O*-hexoside *O*-deoxyhexoside, and taxifolin-3-*O*-rhamnoside) were identified for the first time in SC-CO_2_ extracts of *R. acicularis*, *R. amblyotis*, and *R. rugosa*.

Anthocyanins are water-soluble pigments that turn red, purple, or blue depending on pH. They are also flavonoids but are synthesized via the phenylpropanoid pathway. Anthocyanins are found in all plant matrices, including leaves, stems, and roots, with particularly high concentrations in flowers and fruits. Anthocyanidins are the main structures of anthocyanins. Anthocyanidins consist of an aromatic ring A linked to a heterocyclic ring C containing oxygen, which is also linked via a carbon–carbon bond to a third aromatic ring B [49]. Many researchers have noted that glycoside derivatives of three unmethylated anthocyanidins (pelargonidin, cyanidin, and delphinidin) are the most common in nature, occurring in 69% of fruits, 50% of flowers, and 80% of pigmented leaves [50]. Six anthocyanidins are most commonly found in plants: delphinidin, pelargonidin, malvidin, cyanidin, peonidin, petunidin, and malvidin. Many anthocyanins have sugar residues acylated with aromatic or aliphatic acids [51]. In our studies, we were able to identify anthocyanins belonging to the following groups: delphinidin, pelargonidin, cyanidin, and peonidin—a total of 24 compounds from SC-CO_2_-extracts of *R. acicularis*, *R. amblyotis*, and *R. rugosa*.

## 5. Conclusions

*Rosa* species contain many polyphenolic components and components of other chemical groups that have valuable biological activities. SC-CO_2_ extraction of *R. acicularis*, *R. amblyotis*, and *R. rugosa* was successfully carried out by the team of authors; certain extraction conditions were selected. The extracts obtained showed both a high content of polyphenolic composition and a high content of the saponin group. Tandem mass spectrometry (HPLC-ESI—ion trap) was used to detect target analytes. Mass spectrometric data were recorded on an ion trap equipped with an ESI source in negative and positive ion mode. A four-stage ion separation mode is implemented. Two hundred and eighty-seven different biologically active compounds were found in SC-CO_2_ extracts of *R. acicularis*, *R. amblyotis*, and *R. rugosa*. For the first time, forty-eight chemical constituents from the polyphenol group (15 flavones, 14 flavonols, 4 flavan-3-ols, 3 flavanones, 1 phenylpropanoid, 2 gallotannins, 1 ellagitannin, 4 phenolic acids, 1 dihydrochalcone, and 3 coumarins) were identified in supercritical extracts of *Rosa acicularis*, *Rosa amblyotis*, and *Rosa rugosa*. Our research has shown for the first time the acceptability and effectiveness of the supercritical extraction method in extracting the largest number of chemical compounds of both the polyphenol group and compounds of other groups, which can be a start in improving applied food and pharmaceutical technologies.

## Figures and Tables

**Table 1:** The global yield of extract (mg/100 mg) after SC-CO_2_ extraction of berries of three Rosa species.

Species	Temperature °C /Pressure (Bar)	50	100	150	200	250	300	350
*R. acicularis*	31	0.29 ± 0.012 u	1.23 ± 0.058 r	1.86 ± 0.058 pq	2.03 ± 0.058 k–q	2.30 ± 0.01 f–l	2.66 ± 0.115 Za–d	1.96 ± 0.057 m–q
*R. amblyotis*		0.29 ± 0.01 u	1.16 ± 0.058 r	1.83 ± 0.11 q	1.93 n–q	2.26 ± 0.058 f–m	2.56 ± 0.058 a–f	2.23 ± 0.058 g–n
*R. rugosa*		0.26 ± 0.058 u	1.23 ± 0.058 r	1.93 ± 0.058 n–q	2.10 ± 0.1 j–q	2.33 ± 0.058 e–k	2.63 ± 0.058 Za–e	2.26 ± 0.058 f–m
*R. acicularis*	40	0.43 ± 0.059 Tu	1.96 ± 0.058 m–q	2.13 ± 0.055 i–q	2.36 ± 0.1 d–j	2.73 ± 0.05 X–Za–c	2.93± 0.052 T–Z	20 ± 0.058 l–q
*R. amblyotis*		0.76 ± 0.25 s	1.83 ± 0.058 q	2.16 ± 0.058 h–p	2.36 ± 0.058 d–j	2.70 ± 0.0 Yza–c	2.90 ± 0.0 U–Z	2.50 ± 0.0 b–g
*R. rugosa*		1.30 ± 0.3 r	1.90 ± 0.0 o–q	2.10 ± 0.0 j–q	2.30 ± 0.0 f–l	2.70 ± 0.0 Yza–c	2.90 ± 0.0 U–Z	2.50 ± 0.0 b–g
*R. acicularis*	45	0.70± 0.0 st	2.70 ± 0.1 Yza–c	2.56 ± 0.057 a–f	3.30 ± 0.1 RS	3.06 ± 0.0577 S–W	3.06 ± 0.058 S–W	30 ± 0.1 S–Y
*R. amblyotis*		0.70 ± 0.0 st	2.70 ± 0.0 Yza–c	2.50 ± 0.0 b–g	3.30 ± 0.0 RS	30 ± 0.0 S–Y	3.06 ± 0.058 S–W	3.03 ± 0.058 S–X
*R. rugosa*		1.43 ± 0.252 r	2.70 ± 0.0 Yza–c	2.50 ± 0.0 b–g	3.30 ± 0.0 RS	30 ± 0.0 S–Y	3.10 ± 0.0 S–V	3.13 ± 0.058 S–V
*R. acicularis*	50	2.50 ± 0.0 b–g	3.20 ± 0.0 RSTU	4.03 ± 0.058 M–O	50 ± 0.1 FG	3.96 ± 0.57 N–P	30 ± 0.1 S–Y	3.06 ± 0.11 S–W
*R. amblyotis*		2.56 ± 0.058 a–f	3.26 ± 0.058 RS	4.30 ± 0.10 J–M	4.56 ± 0.058 IJ	4.46 ± 0.058 I–K	3.13 ± 0.115 S–V	3.20 ± 0.0 R–U
*R. rugosa*		2.20 ± 0.265 g–o	4.06 ± 0.115 L–O	4.30 ± 0.0 J–M	4.50 ± 0.0 IJ	4.50 ± 0.0 IJ	3.50 ± 0.0 QR	4.10 ± 0.0 L–O
*R. acicularis*	55	2.66 ± 0.05 Za–d	3.70 ± 0.10 PQ	4.43 ± 0.11 I–K	6.16 ± 0.05 B–D	5.20 ± 0.10 E–G	5.20 ± 0.0 E–G	3.96 ± 0.059 N–P
*R. amblyotis*		2.56 ± 0.058 a–f	3.70 ± 0.0 PQ	4.56 ± 0.058 IJ	5.46 ± 0.058 E	5.23 ± 0.058 E–G	5.20 ± 0.0 E–G	3.83 ± 0.115 OP
*R. rugosa*		2.46 ± 0.231 b–h	3.70 ± 0.0 PQ	4.36 ± 0.115 I–L	5.50 ± 0.0 E	5.20 ± 0.0 E–G	5.20 ± 0.0 E–G	5.10 ± 0.0 FG
*R. acicularis*	60	2.43 ± 0.12 c–i	3.10 ± 0.0 S–V	3.93 ± 0.057 N–P	4.50 ± 0.10 IJ	50 ± 0.0 FG	4.36 ± 0.057 I–L	3.50 ± 0.10 QR
*R. amblyotis*		2.43 ± 0.058 c–i	3.16 ± 0.058 S–U	5.06 ± 0.057 FG	5.30 ± 0.0 EF	6.33 ± 0.06 BC	60 ± 0.0 D	3.50 ± 0.0 QR
*R. rugosa*		2.46 ± 0.058 b–h	3.10 ± 0.0 S–V	60 ± 0.0 D	6.7 ± 0.0 A	6.46 ± 0.058 AB	5.93 ± 0.058 D	4.96 ± 0.231 GH
*R. acicularis*	70	2.30 ± 0.9 f–l	2.83 ± 0.058 V–Za	3.83 ± 0.058 OP	3.90 ± 0.10 N–P	3.06 ± 0.11 S–W	3.23 ± 0.057 R–T	2.93 ± 0.057 T–Z
*R. amblyotis*		2.66 ± 0.153 Za–d	2.83 ± 0.115 V–Za	3.86 ± 0.058 N–P	3.93 ± 0.153 N–P	5.03 ± 0.153 FG	3.93 ± 0.058 N–P	3.23 ± 0.252 R–T
*R. rugosa*		2.76 ± 0.058 W–Zab	2.90 ± 0.1 U–Z	40 ± 0.1 M–P	4.93 ± 0.153 GH	6.10 ± 0.1 CD	4.66 ± 0.058 HI	4.16 ± 0.058 K–N

Note: The values presented are means (n = 3) ± standard deviation. Different letters indicate significant differences among the mean values.

**Table 2:** Jaccard Index for three varieties of wild *Rosa*.

	*Rosa acicularis* (185)	*Rosa amblyotis* (97)	*Rosa rugosa* (94)
*Rosa acicularis* (185)	--	76 0.3689	75 0.3676
*Rosa amblyotis* (97)	76 0.3689	--	42 0.2819
*Rosa rugosa* (94)	75 0.3676	42 0.2819	--

**Table 3:** Jaccard index for three varieties of wild *Rosa*.

	*Rosa acicularis* (22)	*Rosa amblyotis* (20)	*Rosa rugosa* (7)
*Rosa acicularis* (22)	--	13 0.4483	7 0.3182
*Rosa amblyotis* (20)	13 0.4483	--	4 0.1739
*Rosa rugosa* (7)	7 0.3182	4 0.1739	--

## Data Availability

Data are contained within the article.

## Appendix A

**Table A1:** Chemical compounds identified from the SC-CO_2_ extracts of *R. acicularis*, *R. amblyotis*, and *R. rugosa* in positive and negative ionization modes by HPLC-ion trap-MS/MS.

	Class of Compounds	Identification	Formula	Calculated Mass	Observed Mass [M − H]^−^	Observed Mass [M + H]^+^	MS/MS Stage 1 Fragmentation	MS/MS Stage 2 Fragmentation	MS/MS Stage 3 Fragmentation	References
	Polyphenolic Compounds									
**1**	7-hydroxyisoflavone	Formononetin [Biochanin B; Formononetol]	C_16_H_12_O_4_	268.2641		269	251; 137	233	150	*Dracocephalum jacutense* [16]; *Medicago varia* [17]; *Maackia amurense* [18]; Chinese herbal formula Jian-Pi-Yi-Shen pill [19]
**2**	Flavone	Apigenin [5,7-Dixydroxy-2-(40Hydroxyphenyl)-4H-Chromen-4-One]	C_15_H_10_O_5_	270.2369		271	253; 211; 136	225; 179; 134		*Inula gaveolens* [25]; *Lonicera henryi* [26]; *Ribes meyeri* [27]; *Andean blueberry* [28]
**3**	Flavone	Acacetin [Linarigenin; Buddleoflavonol] *	C_16_H_12_O_5_	284.2635		285	267; 211	187		*Triticum aestivum* [20]; *Mentha* [21]; *Mexican lupine* species [22]
**4**	Flavone	Calycosin [3′-Hydroxyformononetin] *	C_16_H_12_O_5_	284.2635		285	253; 235; 217	217; 191; 155	133	Chinese herbal formula Jian-Pi-Yi-Shen pill [19]
**5**	Flavone	Prunetin [Padmakastein]	C_16_H_12_O_5_	284.2635		285	253; 235; 217	217; 191; 155	133	*Triticum aestivum* [20]; *Rosa rugosa* [52]
**6**	Flavone	Luteolin [Tetrahydroxyflavone]	C_15_H_10_O_6_	286.2363		287	269; 241; 227	153		*Inula gaveolens* [25]; *Lonicera henryi* [26]; *Ribes meyeri* [27]; *Rosa rugosa* [52]; *Jatropha* [53]
**7**	Flavone	Diosmetin [Luteolin 4′-Methyl Ether; Salinigricoflavonol] *	C_16_H_12_O_6_	300.2629		301	286; 259	178; 136	150; 121	*Inula viscosa* [54]
**8**	Flavone	Chrysoeriol [Chryseriol]	C_16_H_12_O_6_	300.2629		301	286; 259	178; 136	150; 121	*Mentha* [21]; *Mexican lupine* species [22]
**9**	Pentahydroxyflavone	Herbacetin [3,5,7,8-Tetrahydroxy-2-(4-hydro-xyphenyl)-4H-chromen-4-one]	C_15_H_10_O_7_	302.2357		303	203; 165	221; 175	202	*Triticum aestivum* [20]; *Lonicera caerulea* [55]
**10**	Flavone	Cirsimaritin [Scrophulein; 4′,5-Dihydroxy-6,7-Dimethoxyflavone; 7-Methylcapillarisin]	C_17_H_14_O_6_	314.2895		315	300	272; 168; 135	140	*Artemisia annua* [23]; *Rosmarinus officinalis* [24]; *Medicago varia* [17]
**11**	Flavone	Dihydroxy-dimethoxy(iso)flavone	C_17_H_14_O_6_	314.2895		315	300	272; 168; 135	140	*Rosmarinus officinalis* [24]; *Medicago varia* [17]
**12**	Flavone	Odoratin *	C_17_H_14_O_6_	314.2895		315	300	255; 215; 136	168; 133	*Astragali radix* [56,57]
**13**	Flavone	Salvigenin *	C_18_H_16_O_6_	328.3160	327		312; 164	283; 149	268; 235; 150	*Dracocephalum palmatum* [58]; *Ocimum* [59]
**14**	Flavone	Jaceosidin [5,7,4′-trihydroxy-6′,5′-dimethoxyflavone] *	C_17_H_14_O_7_	330.2889		331	303; 203	203; 275	222	*Artemisia argyl* [60]; *Mentha* [21]
**15**	Flavone	Dimethoxy-trihydroxy(iso)flavone	C_17_H_14_O_7_	330.2889		331	303; 203	203; 275	222	*Jatropha* [53]
**16**	Flavone	5,6,4′-Trihydroxy-7,8-dimethoxyflavone	C_17_H_14_O_7_	330.2889		331	313; 203	285	267; 241	*Mentha* [21]; *F. glaucescens*; *F. herrerae* [61]
**17**	Flavone	Cirsiliol	C_17_H_14_O_7_	330.2889	329		229; 311	209; 125	209	*Juglans mandshurica* [62]; *Ocimum* [59]; *Inula viscosa* [54]; *Medicago varia* [17]
**18**	Flavone	Nevadensin *	C_18_H_16_O_7_	344.3154		345	312; 270; 179	284; 269	269; 255; 182; 134	*Mentha* [21]; *Ocimum* [59]; *Medicago varia* [17]
**19**	Flavone	Cirsilineol [Eupatrin; Fastigenin; Cirsileneol] *	C_18_H_16_O_7_	344.3154		345	312; 270; 179	284; 269	269; 255; 182; 134	*Ocimum* [59]; *Medicago varia* [17]
**20**	Flavone	5,6-Dihydroxy-7,3′,4′ -trimethoxyflavone	C_18_H_16_O_7_	344.3154		345	312; 270; 179	284; 269	269; 255; 182; 134	*Mentha* [21]; *A. cordifolia* [61]
**21**	Flavone	5,6-Dihydroxy-7,8,3′,4′-tetramethoxyflavone	C_19_H_18_O_8_	374.3414		375	342; 317	314; 299	299; 284; 180; 151	*Mentha* [21]
**22**	Flavone	Tetrahydroxyflavanone pentoside	C_20_H_20_O_10_	420.3668	419		285	255	255; 227	*F. glaucescens* [61]
**23**	Flavone	Eriodictyol-*O*-pentoside	C_20_H_20_O_10_	420.3668	419		285	255	255; 227	*Andean blueberry* [28]
**24**	Flavone	Tetrahydroxy-methoxyflavone-*O*-pentoside	C_20_H_16_O_12_	448.3338		449	303	303; 257	229; 201	*Carpinus betulus* [63]
**25**	Flavone	Luteolin 7-O-glucoside [Cynaroside; Luteoloside]	C_21_H_20_O_11_	448.3769		449	287	287; 241; 213		*Vaccinium macrocarpon* [29]; *Lonicera henryi* [26]
**26**	Flavone	Luteolin-O-hexoside	C_21_H_20_O_11_	448.3769		449	287	287; 241; 213		*Lepechinia* [64]; *Ribes meyeri* [27]; *Chilean currants* [65]
**27**	Flavone	Luteolin-*O*-glucuronide	C_21_H_18_O_12_	462.3604		463	287	241; 165	241; 213; 157	*Andean blueberry* [28]
**28**	Glucosyloxyflavone	Scutellarin [Breviscapin; Scutellarin B; Scutellarin-7-glucuronide] *	C_21_H_18_O_12_	462.3604		463	287	241; 165	213; 157	*Perilla frutescens* [66]
**29**	Flavone	Chrysoeriol 6-*O*-hexoside *	C_22_H_22_O_11_	462.4036		463	445	427	409; 213	*Triticum aestivum* L. [67]
**30**	Flavone	Chrysoeriol 6-C-glucoside [Isoscoparin; Isoscoparine] *	C_22_H_22_O_11_	462.4035		463	445	427	409; 213	*Triticum aestivum* L. [67]
**31**	Flavone	Chrysoeriol 8-C-glucoside [Scoparin] *	C_22_H_22_O_11_	462.4035		463	445	427	409; 213	*Mexican lupine* species [22]; *Citrus sinensis* [68]
**32**	Flavone	Apigenin-7-O-rutinoside [Isorhoifolin]	C_27_H_30_O_14_	578.5187		579	417; 291	287	269; 255	*Dracocephalum palmatum* [58]; *Mentha* [21]
**33**	Flavone	Apigenin 8-C-deoxyhexoside-7-O-glucoside	C_27_H_30_O_14_	578.5187		579	417; 291; 163	287	269; 255	*Passiflora incarnata* [69]
**34**	Flavone	Chrysin 6-C-[2″-*O*-glucoside] -glucoside *	C_27_H_30_O_14_	578.5187		579	417; 291	287; 369	269; 255	*Passiflora incarnata* [69]
**35**	Flavone	Chrysin di-*O*-glucoside *	C_27_H_30_O_14_	578.5187		579	417; 291	287	269; 255	*Passiflora incarnata* [69]
**36**	Flavone	Lonicerin [Luteolin-7-O-Rhamnoside; Veronicastroside; Scolymoside; Luteolin-7-Rhamnoglucoside] *	C_27_H_30_O_15_	594.5181		595	449; 287	287	287; 153	*Lonicera caerulea* [55]
**37**	Flavone	Luteolin *7-O-*(*6-O*-rhamnosyl-hexoside)	C_27_H_30_O_15_	594.5181		595	287	287; 241		*Lonicera henryi* [26]
**38**	Hydroxy(iso)flavone	2′-Hydroxygenistein *C*-diglucoside malonylated	C_30_H_32_O_19_	696.5637	695		487	469; 427; 353		*Mexican lupine* species [22]
**39**	Flavonol	Kaempferol	C_15_H_10_O_6_	286.2363		287	227; 189; 153; 110	152		*Inula gaveolens* [25]; *Juglans mandshurica* [62]; *Ribes meyeri* [27]; *Andean blueberry* [28]
**40**	Flavonol	Rhamnocitrin	C_16_H_12_O_6_	300.2629		301	283	199		*Astragali radix* [56]; *Lonicera caerulea* [55]
**41**	Flavonol	Kaempferide [4′-O-Methylkaempferol]	C_16_H_12_O_6_	300.2629		301	286; 259	178; 136	150; 121	*Triticum aestivum* [20]; *Ocimum* [59]; *Ribes meyeri* [27]; *Juglans mandshurica* [62]
**42**	Flavonol	Isokaempferide [3-O-Methylkaempferol]	C_16_H_12_O_6_	300.2629		301	286; 259	178; 136	150; 121	*Rosa rugosa* [52]
**43**	Flavonol	Quercetin	C_15_H_10_O_7_	302.2357		303	303; 257; 165	229	201	*Ribes meyeri* [27]; *Capsicum annuum* [70]
**44**	Flavonol	Hesperitin [Hesperetin]	C_15_H_10_O_7_	302.2357		303	285; 165	257; 173	229; 201	*Andean blueberry* [28]; *Rosmarinus officinalis* [24]
**45**	Flavonol	Dihydroquercetin (Taxifolin; Taxifoliol)	C_15_H_12_O_7_	304.2516		305	277	187		*Juglans mandshurica* [62]; *Camellia kucha* [71]; *Andean blueberry* [28]
**46**	Flavonol	Isorhamnetin [Isorhamnetol; Quercetin 3′-Methyl ether; 3-Methylquercetin]	C_16_H_12_O_7_	316.2623		317	271; 207; 179; 151	151		*Inula viscosa* [54]; *Vaccinium macrocarpon* [72]; *Andean blueberry* [28]; *Lonicera caerulea* [55]; *Ribes dikuscha* [73]; *Rosa rugosa* [52]; *Rosmarinus officinalis* [24]
**47**	Flavonol	Rhamnetin I *	C_16_H_12_O_7_	316.2623		317	271; 207; 179; 151	151		*Inula viscosa* [54]; *Inula gaveolens* [25]; *Polygala sibirica* [74]; *Ribes dikuscha* [73]
**48**	Flavonol	Rhamnetin II *	C_16_H_12_O_7_	316.2623		317	271; 207; 179; 151	151		*Inula gaveolens* [25]; *Polygala sibirica* [74]
**49**	Flavonol	Myricetin	C_15_H_10_O_8_	318.2351		319	273; 219	191	209	*Juglans mandshurica* [62]; *Andean blueberry* [28]
**50**	Flavonol	Ampelopsin [Dihydromyricetin; Ampeloptin]	C_15_H_12_O_8_	320.251		321	273; 239; 219	191	173	*Juglans mandshurica* [62]
**51**	Flavonol	Kaempferol 3-O-pentoside	C_20_H_18_O_10_	418.3509		419	257	153		*Andean blueberry* [28]; Cranberry [75]; *Rosa canina* [76]; *Ribes dikuscha* [73]
**52**	Flavonol	Avicularin (Quercetin 3-Alpha-L-Arabinofuranoside; Avicularoside)	C_20_H_18_O_11_	434.3503	433		301	179	151	*Rosa rugosa* [77]; *Ribes meyeri* [27]; Cranberry [78]; *Juglans mandshurica* [62]
**53**	Flavonol	Quercetin-3-*alpha*-xylopyranoside [Quercetin 3-*O*-xylopyranoside]	C_20_H_18_O_11_	434.3503	433		301	179	151	*Vaccinium macrocarpon* [72]; Cranberry [78]
**54**	Flavonol	Quercetin 3-*O*-pentoside	C_20_H_18_O_11_	434.3503	433		301	179	151	*Rosa canina* [76]; *Ribes meyeri* [27]
**55**	Flavonol	Quercetin 3-D-xyloside [Reynoutrin]	C_20_H_18_O_11_	434.3503		435	303	257; 165	229; 201	*Juglans regia* [79]; *Embelia* [80]; Cranberry [75]
**56**	Flavonol	Quercetin-3-O-arabinoside [Guaijaverin; Guajaverin; Quercetin-3-Arabinopyranoside]	C_20_H_18_O_11_	434.3503	433		301	179	151	*Juglans regia* [79]; *Lonicera henryi* [26]; Cranberry [78]; Cranberry [75]; *Embelia* [80]
**57**	Flavonol	Taxifolin-O-pentoside [Dihydroquercetin pentoside]	C_20_H_20_O_11_	436.371	435		303	285; 125	241; 175	*A. cordifolia* [61]; *Rosa davurica* [81]; *Chilean currants* [65]
**58**	Flavonol	Taxifolin 3-O-arabinofuranoside [(+)-taxifolin 3-O-alpha-D-arabinopyranoside] *	C_20_H_20_O_11_	436.371	435		303	285; 125	241; 175	Strawberry [82]
**59**	Flavonol	Quercitrin [Quercetin 3-O-rhamnoside; Quercetrin]	C_21_H_20_O_11_	448.3769		449	303	227	229; 201	Honey [83]; *Carpinus betulus* [63]; *Inula viscosa* [54]; *Juglans mandshurica* [62]; *Vaccinium macrocarpon* [29,72]; Cranberry [75,78]
**60**	Flavonol	Quercetin 3-O-deoxyhexoside	C_21_H_20_O_11_	448.3770		449	303	227	229; 201	*Myrtle* [30]; *Stevia rebaudiana* [84]
**61**	Flavonol	Isorhamnetin-3-O-arabinoside *	C_21_H_20_O_11_	448.3769	447		315; 284; 235	300		*Vitis vinifera* [85]
**62**	Flavonol	Isorhamnetin 3-*O*-pentoside *	C_21_H_20_O_11_	448.3769	447		315; 284; 235	300		*Andean blueberry* [28]; Cranberry [75]
**63**	Flavonol	Astragalin [Kaempferol 3-*O*-glucoside; Kaempferol-3-Beta-Monoglucoside; Astragaline] *	C_21_H_20_O_11_	448.3769		449	287	287; 241; 213		*Juglans mandshurica* [62]; *Ribes meyeri* [27]
**64**	Flavonol	Kaempferol-3-*O*-galactoside	C_21_H_20_O_11_	448.3769	447		285	287; 241; 213		*Triticum aestivum* [20]; *Vitis vinifera* [85]; *Ribes nigrum* [76]
**65**	Flavonol	Kaempferol-3-*O*-hexoside	C_21_H_20_O_11_	448.3769		449	287	287; 241; 213		*Andean blueberry* [28]
**66**	Flavonol	Kaempferol-*C*-hexoside	C_21_H_20_O_11_	448.3769	447		285; 255; 151	255	227	*Vaccinium macrocarpon* [29]; *Ribes dikuscha*, *Ribes pauciflorum* [73]
**67**	Flavonol	Methylquercetin-pentoside	C_21_H_20_O_11_	448.3769		449	317	285	257	*Vaccinium macrocarpon* [72]
**68**	Flavonol	Dihydrokaempferol 3-O-glucoside	C_21_H_22_O_11_	450.3928		451	289	271; 163; 127	229	*Punica granatum* [86]
**69**	Flavonol	Dihydrokaempferol-*O*-hexoside *	C_21_H_22_O_11_	450.3928		451	289; 273	271; 163; 127	229; 201	*Punica granatum* [86]
**70**	Flavonol	Aromadendrin O-hexoside *	C_21_H_22_O_11_	450.3928		451	289	271; 163; 127	229	*Andean blueberry* [28]
**71**	Flavonol	Kaempferol-3-*O*-glucuronide	C_21_H_18_O_12_	462.3604		463	287	241; 165	241; 213; 157	*Vitis vinifera* [85]; Strawberry [87]; *Ribes fragrans* [43]; *A. cordifolia*; *G. linguiforme* [61]; *Rosa canina* [76]
**72**	Flavonol	Kaempferol hexuronide	C_21_H_18_O_12_	462.3604		463	287	241; 165	241; 213; 157	*Hibiscus rosa sinensis* [88]
**73**	Flavonol	Quercetin-3-O-hexoside	C_21_H_20_O_12_	464.3763		465	303	257; 165	229; 201	Cranberry [78]; *Ribes dikuscha* [73]; *Stevia rebaudiana* [84]
**74**	Flavonol	Hyperoside (Quercetin 3-O-galactoside; Hyperin)	C_21_H_20_O_12_	464.3763		465	303	257; 165	229; 201	*Vaccinium macrocarpon* [29,72]; Cranberry [75]; *Andean blueberry* [28]; *Camelia kucha* [71]
**75**	Flavonol	Quercetin 3-glucoside [Isoquercetin; Isoquercitrin; Hirsutrin; Quercetin-3-O-Glucopyranoside; 3-Glucosylquercetin]	C_21_H_20_O_12_	464.3763		465	303	257; 165	229; 201	*Ribes meyeri* [27]; *Lonicera henryi* [26]; Cranberry [75]; *Andean blueberry* [28]; *Ribes fragrans* [43]
**76**	Flavonol	Quercetin 7-O-glucoside [Quercetin-7-O-Beta-Glucopyranoside]	C_21_H_20_O_12_	464.3763		465	303	257; 165	229; 201	*Passiflora incarnata* [69]; *Capsicum annuum* [70]
**77**	Flavonol	Taxifolin-3-*O*-glucoside *	C_21_H_22_O_12_	466.3922	465		285; 213	241; 217; 149	241; 197	*Vitis vinifera* [85]
**78**	Flavonol	Taxifolin-3-*O*-hexoside [Dihydroquercetin-3-O-hexoside] *	C_21_H_22_O_12_	466.3922	465		285; 213	241; 217; 149	241; 197	*Andean blueberry* [28]; *Chilean currants* [65]
**79**	Flavonol	Quercetin-3-O-glucuronide [Miquelianin]	C_21_H_18_O_13_	478.3598		479	303	257; 229	201	*Vitis vinifera* [85]; *Rosa canina* [76]; Strawberry [87]
**80**	Flavonol	Quercetin-hexuronide *	C_21_H_18_O_13_	478.3598		479	303	257; 165	229; 145	*Hibiscus rosa sinensis* [88]
**81**	Flavonol	Myricetin-3-*O*-galactoside	C_21_H_20_O_13_	480.3757		481	319	273; 245; 181; 137	245; 227; 157	*Juglans mandshurica* [62]; *Vitis vinifera* [85]; *Vaccinium macrocarpon* [29,72]; Cranberry [75]
**82**	Flavonol	Myricetin-3-*O*-glucoside	C_21_H_20_O_13_	480.3757	479		316; 179	271; 179	243	*Vitis vinifera* [85]; *Camellia kucha* [71]
**83**	Flavonol	Myricetin 3-*O*-hexoside	C_21_H_20_O_13_	480.3757	479		316; 179	271; 179	243	*Carpinus betulus* [63]; *Myrtle* [30]; *Andean blueberry* [28]; Cranberry [78]; *Chilean currants* [65]
**84**	Flavonol	Quercetin 3-*O*-malonylglucoside	C_24_H_22_O_15_	550.4225		551	303	257; 165	229; 201; 161	*Mexican lupine* species [22]; *A. cordifolia* [61]; Strawberry [87]
**85**	Flavonol	Quercetin 3-*O*-(*6*″-*O*-malonyl)hexoside	C_24_H_22_O_15_	550.4225		551	303	257; 165	229; 201; 161	Bee-pollen [89]
**86**	Flavonol	Kaempferol 3-*O*-rhamnosyl-galactoside	C_27_H_30_O_15_	594.5181		595	287; 165	287		Pear [90]
**87**	Flavonol	Kaempferol 3-*O*-(*6*-*O*-rhamnosyl-glucoside)	C_27_H_30_O_15_	594.5181		595	287	241; 213; 165	213; 141	*Mexican lupine* species [22]; *Lonicera henryi* [26]
**88**	Flavonol	Kaempferol rhamnosyl glucoside	C_27_H_30_O_15_	594.5181		595	287; 219; 165	241; 165	213	*Tilla tomentosa* [76]
**89**	Flavonol	Kaempferol 3-*O*-deoxyhexoside-hexoside	C_27_H_30_O_15_	594.5181		595	287; 219; 165	241; 165	213	*Stevia rebaudiana* [84]; *Hibiscus rosa sinensis* [88]; *Rosa rugosa* [91]
**90**	Flavonol	Kaempferol 7-*O*-neohesperidoside	C_27_H_30_O_15_	594.5181		595	287; 219; 165	241; 165	213	Honey [83]
**91**	Flavonol	Kaempferol-3-*O*-coumaroylhexoside	C_27_H_30_O_15_	594.5181		595	287	213		Strawberry [87]
**92**	Flavonol	Kaempferol 3-*O*-rutinoside	C_27_H_30_O_15_	594.5181		595	287	241; 213; 165	213; 141	*Ribes meyeri* [27]; *Spondias purpurea* [92]
**93**	Flavonol	Kaempferol 3-*O*-neohesperidoside	C_27_H_30_O_15_	594.5181		595	287	241; 213; 165	213; 141	*Rosa rugosa* [1]
**94**	Flavonol	Quercetin-3-hydroxyl-3-methylglutaroyl(HMG)-glucoside	C_28_H_32_O_15_	608.5447		609	303	285; 229; 165	201; 145	*Rubus ulmifolius* [93]
**95**	Flavonol	Rutin (Quercetin 3-*O*-rutinoside)	C_27_H_30_O_16_	610.5175		611	303	303; 257; 165	229	*Ribes meyeri* [27]; *Ribes magellanicum* [64]; *Lonicera henryi* [26]; *Spondias purpurea* [92]
**96**	Flavonol	Quercetin-O-rhamnoside-O-hexoside	C_27_H_30_O_16_	610.5175		611	303	303; 257; 165	229	*Spondias purpurea* [92]
**97**	Flavonol	Quercetin rhamnosyl glucoside	C_27_H_30_O_16_	610.5175		611	303	303; 257; 165		*Mexican lupine* species [22]; *Tilla tomentosa* [76]; *Ribes dikuscha* [73]
**98**	Flavonol	Quercetin-3-O-rhamnoside-7-O-glucoside	C_27_H_30_O_16_	610.5175		611	303	303; 257; 165	229	*Mexican lupine* species [22]; *Tilla tomentosa* [76]
**99**	Flavonol	Quercetin 7-O-rutinoside	C_27_H_30_O_16_	610.5175		611	303	303; 257; 165	229	Pear [90]; *Vitis vinifera* [85]
**100**	Flavonol	Quercetin *O*-hexoside *O*-deoxyhexoside *	C_27_H_30_O_16_	610.5175		611	303	303; 257; 165	229	*Andean blueberry* [28]
**101**	Flavonol	Bioquercetin [Quercetin-3-*O*-robinobioside] *	C_27_H_30_O_16_	610.5175		611	303	303; 257; 165	229	*Aspalathus linearis* [94]
**102**	Flavonol	Quercetin-O-(O-galloyl) hexoside	C_28_H_24_O_16_	616.4806		617	599; 303	257	229	*Juglans regia* [79]; *Myrtle* [30]
**103**	Flavonol	Quercetin galloyl-glucoside	C_28_H_24_O_16_	616.4806		617	303	257; 165	229; 158	*Rosa canina* [76]
**104**	Flavonol	Quercetin 3,4′-di-O-beta-glucopyranoside [Quercetin diglucoside]	C_27_H_30_O_17_	626.5169		627	465; 303	303; 257; 165	229	*Rosa rugosa* [95]; *Ribes nigrum* [76]; *Medicago varia* [17]
**105**	Flavonol	Quercetin-*O*-dihexoside	C_27_H_30_O_17_	626.5169		627	465; 304	303; 257; 166	230	*Inula viscosa* [54]; *Chilean currants* [65]; *Andean blueberry* [28]; *Medicago varia* [17]; *Hibiscus rosa sinensis* [88]
**106**	Flavonol	Quercetin-3,7-di-*O*-hexoside	C_27_H_30_O_17_	626.5169		627	465; 305	303; 257; 167	231	Bee-pollen [89]
**107**	Flavonol	Herbacetin-di-O-glucoside	C_27_H_30_O_17_	626.5169		627	465; 306	303; 257; 168	232	*Rhodiola rosea* [96]
**108**	Flavonol	Quercetin-3-O-xylosylrhamnosyl glucoside	C_33_H_40_O_19_	740.6593	739		587; 493; 269	285; 243		*Ribes meyeri* [27]
**109**	Flavonol	Kaempferol-3-O-alpha-L-rhamnopyranosyl-(1->3)-(4-O-acetyl-alpha-L-rhamnopyranosyl)-(1->6)-beta-D-galactopyranoside	C_34_H_22_O_22_	782.5253		783	303; 463	285	257	*Actinidia valvata* [97]
**110**	Flavan-3-ol	Afzelechin *	C_15_H_14_O_5_	274.2687		275	245; 175	157		*Camellia kucha* [71]; *Pelargonium endlicherianum* [98]
**111**	Flavan-3-ol	Epiafzelechin [(epi)Afzelechin] *	C_15_H_14_O_5_	274.2687		275	245; 219; 175	215; 193; 175; 157; 127	175; 157; 145	*A. cordifolia*; *F. glaucescens*; *F. herrerae* [61]
**112**	Flavan-3-ol	Catechin	C_15_H_14_O_6_	290.2681		291	261; 273; 231; 200	191; 173; 143	161; 143	*Ribes meyeri* [27]; *Ribes magellanicum* [65]
**113**	Flavan-3-ol	(Epi)-catechin	C_15_H_14_O_6_	290.2681		291	261; 273; 231; 200	191; 173; 143	161; 143	*Andean blueberry* [28]; *C. edulis* [61]; *Radix polygoni multiflori* [99]
**114**	Flavan-3-ol	Gallocatechin [+(−)Gallocatechin] *	C_15_H_14_O_7_	306.2675		307	287; 153	259; 147	149	*Carpinus betulus* [63]; *G. linguiforme* [61]; *Ribes meyeri* [27]; *Embelia* [80]
**115**	Flavan-3-ol	(Epi)-Gallocatechin *	C_15_H_14_O_7_	306.2675		307	287; 153	259; 147	149	*Juglans regia* [79]; *Carpinus betulus* [63]; *Myrtle* [30]; *Ribes meyeri* [27]; *Ribes magellanicum* [65]; *G. linguiforme* [61]; *Camellia kucha* [71]
**116**	Flavan-3-ol	(Epi)-catechin derivative 1	C_19_H_22_O_8_	378.3732		379	261; 275; 233	233; 207; 179	179; 165; 151	Pubchem
**117**	Flavan-3-ol	(Epi)-afzelechin derivative	C_18_H_16_O_10_	392.3136		393	275; 375	245; 175	157	*Zostera marina* [45]
**118**	Flavan-3-ol	(Epi)-catechin derivative 2	C_18_H_16_O_11_	408.3130		409	291; 220; 155	272; 231; 187; 159	243; 213; 185	*Lonicera caerulea* [55]
**119**	Flavan-3-ol	(Epi)-catechin derivative 3		424		425	291	261	261; 173	PubChem
**120**	Flavan-3-ol	Catechin glucoside	C_21_H_24_O_11_	452.4087		453	291; 273; 205	273; 165; 123	165; 123	*Glycine soja* [100]
**121**	Flavan-3-ol	(epi)-Catechin glucoside	C_21_H_24_O_11_	452.4087		453	291; 273; 205	273; 165; 123	165; 123	*Cassia abbreviata* [101]
**122**	Flavan-3-ol	(epi)-Catechin O-hexoside	C_21_H_24_O_11_	452.4087		453	291; 273; 205	273; 165; 123	165; 123	*Andean blueberry* [28]
**123**	Dihydroxyflavanone	Pinocembrin [(+)-Pinocembrin; Pihyrochrysin; (2S)-Pinocembrin]	C_15_H_12_O_4_	256.2534		257	259; 227; 197; 163	187; 155		*Ribes triste* [73]
**124**	Tetrahydroxyflavanone	Dihydrokaempferol [Aromadendrin; Katuranin]	C_15_H_12_O_6_	288.2522		289	271; 177; 141	252; 193; 167	193	*Juglans mandshurica* [62]; *Andean blueberry* [28]; *F. glaucescens* [61]; *Camellia kucha* [71]
**125**	Flavanone	Quercetin O-hexoside O-deoxyhexoside [3′,4′,5,7-tetrahydroxy-flavanone] *	C_15_H_12_O_6_	288.2522		289	271; 242; 220; 127	253; 224; 193; 165	193	*Andean blueberry* [28]; *Embelia* [80]; *Rosa rugosa* [52]; *Rosmarinus officinalis* [24]; *Jatropha* [53]
**126**	Flavanone	Hesperitin [Hesperetin] *	C_16_H_14_O_6_	302.2788		303	303; 257; 165	229	201	*Andean blueberry* [28]; *Ribes triste* [73]; *Vitis vinifera* [85]; *Rosmarinus officinalis* [24]
**127**	Flavanone	Taxifolin-3-O-rhamnoside [Dihydroquercetin-3-O-rhamnoside; Astilbin; Isoastilbin; Neoastilbin] *	C_21_H_22_O_11_	450.3928		451	289; 147	243; 215; 153	215; 149	*Juglans mandshurica* [62]; *Vitis vinifera* [85]
**128**	Flavan	Dihydroxy-dimethoxy(iso)flavan	C_16_H_16_O_5_	288.2952		289	271; 177; 141	252; 193; 167	193	*Astragali radix* [56]
**129**	Glucoside flavonoid	Gossypitrin [Grossypitrin; Articularin; Equisporoside; Gossypetin-7-glucoside]	C_21_H_20_O_13_	480.3757		481	301; 241	241; 283; 199	199	*Triticum aestivum* [20]
**130**	Phenylpropanoid (cinnamic alcohol glycoside)	Vimalin *	C_16_H_22_O_7_	326.3417		327	309; 270; 249; 187	291; 235	250	*Medicago varia* [17]
**131**	Gallate ester	Beta-Glucogallin [1-O-Galloyl-Beta-D-Glucose; Galloyl glucose; Monogalloyl glucose]	C_13_H_16_O_10_	332.2601		333	314; 269; 214; 177; 141	269; 235; 197; 152; 136	134	*Ocimum* [59]; Strawberry [82]; *Eucalyptus* [102]; *Rosa canina* [76]; *Rosa acicularis* [81]; *Pelargonium* [98]
**132**	Gallate ester	Gallic acid hexoside	C_13_H_16_O_10_	332.2601		333	314; 269; 214; 177; 141	269; 235; 197; 152; 136	134	*Ribes meyeri* [27]
**133**		Methylgalloyl glucose		346	345		183	168		*Rosa canina* [76]
**134**		Methylgalloyl hexose		346	345		183	168		*Carpinus betulus* [63]
**135**		Galloyl-methylgalloyl hexose		498	497		465; 345	183	168; 124	*Carpinus betulus* [63]
**136**		Trigalloyllevoglucosan	C_27_H_22_O_17_	618.4534		619	303; 191	229; 165	201; 132	Pubchem
**137**		Tri-galloyl hexoside	C_27_H_24_O_18_	636.4687	635		465	313; 295; 235; 168	295; 241; 193; 168	*Carpinus betulus* [63]; *Ribes dikuscha* [73]
**138**		Tri-galloyl glucose	C_27_H_24_O_18_	636.4687	635		465	313; 295; 235; 168	295; 241; 193; 168	*Juglans regia* [79]; *Ribes dikuscha* [73]
**139**	Gallic acid derivative	1,2,3,6-Tetra-O-galloyl-β-D-glucopyranoside [1,2,3,6-Tetra galloyl glucose]	C_34_H_28_O_22_	788.5729	787		617; 573; 465; 295	465; 331; 277; 215	313; 221	*Eucalyptus* [102]
**140**	Gallic acid derivative	1,2,3,4-Tetra-*O*-galloyl-β-D-glucopyranoside	C_34_H_28_O_22_	788.5729	787		617; 573; 465; 295	465; 331; 277; 215	313; 221	Pubchem
**141**	Gallic acid derivative	Tetra-O-galloylhexoside	C_34_H_28_O_22_	788.5729	787		617; 573; 465; 295	465; 331; 277; 215	313; 221	*Carpinus betulus* [63]
**142**	Gallic acid derivative	Tetra-O-galloyl glucose	C_34_H_28_O_22_	788.5729	787		617; 573; 465; 295	465; 331; 277; 215	313; 221	*Carpinus betulus* [63]
**143**	Pro-anthocyanidin	Procyanidin A-type dimer	C_30_H_24_O_12_	576.501		577	425; 245	407; 245; 217	217; 189	*Carpinus betulus* [63]; *Vitis vinifera* [85]; Pear [90]; *Vaccinium macrocarpon* [29,72]
**144**	Pro-anthocyanidin	Procyanidin B1; Procyanidin Dimer B1 [Epicatechin-(4beta->8)-ent-epicatechin]	C_30_H_26_O_12_	578.5202		579	409; 287	287; 257	245; 203	*Vaccinium macrocarpon* [29]; Cranberry [78]; *Andean blueberry* [28]; Strawberry [87]; Cranberry [75]
**145**	Pro-anthocyanidin	Procyanidin B2 [Epicatechin-(4beta->8)-epicatechin]	C_30_H_26_O_12_	578.5202		579	409; 287	287; 257	245; 203	*Punica granatum* [86]; *Glycine soja* [100]; *Vitis vinifera* [85]
**146**	Pro-anthocyanidin	Proanthocyanidin B3 [Proanthocyanidin; Catechin-(4 alpha->8)-catechin]	C_30_H_26_O_12_	578.5202		579	427; 291	409; 301	287; 257	*Punica granatum* [86]; *Vitis vinifera* [85]
**147**	Pro-anthocyanidin	Procyanidin B2 dimethyl gallate [Epicatechin-(4-8)-epicatechin-O-(3,4-dimethyl)-gallate]	C_35_H_34_O_19_	758.6331		759	617; 502; 280	443	160	*F. esculentum* [103,104]
**148**	Anthocyanin	Delphinidin	C_15_H_11_O_7_	303.2436		303	257; 165	229	201	*A. cordifolia* [61]
**149**	Anthocyanin	Delphinidin *O*-pentoside	C_20_H_19_O_11_	435.3583	433		301	179	151	*Myrtle* [30]; *Gaultheria mucronata*; *Gaultheria antarctica* [31]; *Andean blueberry* [28]
**150**	Anthocyanin	Delphinidin 3-*O*-xyloside	C_20_H_19_O_11_	435.3583	433		301	179	151	*Black currant* [105]
**151**	Anthocyanin	Cyanidin-3-*O*-glucoside [Cyanidin 3-*O*-beta-D-Glucoside; Kuromarin; Chrysanthemin]	C_21_H_21_O_11_+	449.3848		450	287	287; 241; 213		*Black currant*, Gooseberry, Chokeberry, Elderberry, *Red currant* [105]; *Ribes magellanicum* [65]; *Berberis ilicifolia*; *Berberis empetrifolia*; *Ribes maellanicum*; *Ribes cucullatum*; *Myrteola nummalaria*; *Gaultheria mucronata*; *Gaultheria antarctica*; *Rubus geoides*; *Fuchsia magellanica* [31]
**152**	Anthocyanin	Cyanidin-3-*O*-hexoside	C_21_H_21_O_11_+	449.3849		450	287	287; 241; 213		*Myrtle* [30]; *Andean blueberry* [28]
**153**	Anthocyanin	Cyanidin-3-*O*-beta-galactoside	C_21_H_21_O_11_	449.3848		450	287	287; 139		*Black soybean* [106]; *Gaultheria mucronata* [31]
**154**	Anthocyanin	Delphinidin 3-*O*-glucoside	C_21_H_21_O_12+_	465.3905		465	303	257; 165	229	*Ribes magellanicum* [65]; *Berberis ilicifolia*; *Berberis empetrifolia*; *Ribes maellanicum*; *Ribes cucullatum*; *Myrteola nummalaria* [31]; *Black currant* [105]
**155**	Anthocyanin	Delphinidin 3-*O*-hexoside	C_21_H_21_O_12+_	465.3905		465	303	257; 165	229; 201	*Myrtle* [30]; *Andean blueberry* [28]
**156**	Anthocyanin	Delphinidin 3-*O*-beta-galactoside	C_21_H_21_O_12+_	465.3905		465	303	257	229	*Vigna angularis* [107]; *Gautheria micronata*; *Gautheria antarctica* [31]
**157**	Anthocyanin	Delphinidin 3-*O*-hexuronide	C_21_H_19_O_13+_	479.3678		479	303	257; 229; 165	201	*Grape vine varieties* [108]
**158**	Anthocyanin	Delphinidin 3-*O*-glucuronide	C_21_H_19_O_13+_	479.3678		479	303	257; 229; 165	201	Vine [109]
**159**	Anthocyanin	Peonidin 3-O-glucoside-4-vinylphenol	C_30_H_27_O_12_	579.5282		579	417; 291	287	269; 255	Vine [109]
**160**	Anthocyanin	Cyanidin 3-*O*-(6-*O*-*p*-coumaroyl)glucoside	C_30_H_27_O_13_	595.533		595	287	287; 241	213	Grape [110]; Vines [109]; *Vitis vinifera* [85]; *Black currant*, Gooseberry [105]
**161**	Anthocyanin	Cyanidin 3-*O*-coumaroyl hexoside	C_30_H_27_O_13_	595.533		595	287	241; 213; 165	213; 141	*Grape vine varieties* [108]
**162**	Anthocyanin	Delphinidin 3-*O*-rutinoside [Tulipanin; Delphinidin 3-Rhamnosyl-Glucoside]	C_27_H_31_O_16_	611.5254		611	465; 303	303; 257; 165	229	*Black currant* [105]; *Berberis ilicifolia*; *Berberis empetrifolia*; *Ribes maellanicum*; *Ribes cucullatum* [31]
**163**	Anthocyanin	Delphinidin 3-*O*-(6-*O*-*p*-coumaroyl) glucoside	C_27_H_31_O_17_	611.5255		611	465; 303	303; 257; 165	229	*Vigna angularis* [107]; Grape [110]; Vines [109]; *Grape vine varieties* [108]
**164**	Anthocyanin	Delphinidin 3-(2″-galloylgalactoside)	C_28_H_25_O_16_	616.4885		617	303	257; 165	229; 201	*Aristotelia chinensis* [111]
**165**	Anthocyanin	Delphinidin-3,5-diglucoside	C_27_H_31_O_17_	627.5248		627	465; 304	303; 257; 166	230	*Vigna angularis* [107]; Grape [110]; *Aristotelia chilensis* [111]
**166**	Anthocyanin	Delphinidin 3,5-dihexoside	C_27_H_31_O_17_	627.5248		627	465; 305	303; 257; 167	231	*F. herrerae* [61]; *Andean blueberry* [28]; *Ribes dikuscha* [73]
**167**	Anthocyanin	Delphinidin-3,5-O-digalactoside	C_27_H_31_O_17_	627.5248		627	465; 306	303; 257; 168	232	*Vigna angularis* [107]
**168**	Anthocyanin	Pelargonidin-3-O-rutinoside-5-O-beta-D-glucoside	C_33_H_41_O_19_	741.6771		741	579; 553; 451; 355; 289	271	229; 187	*Gentiana lutea* [112]; *Iris dichotoma* [113]; *Camellia kucha* [71]
**169**	Anthocyanin	Delphinidin 3-*O*-(6-*O*-*p*-feruloyl)-5-*O*-diglucoside		803.623		804	303	285	257	*Vitis vinifera* [114]
**170**	Anthocyanin	Cyanidin 3-O-(caffeoyl)rutinoside-5-O-glucoside	C_42_H_47_O_22_	903.8219	901		449; 323; 269	269	225	*Iris dichotoma* [113]
**171**	Anthocyanin	Cyanidin 3-(p-coumaroyl)-rutinoside-5-glucoside	C_42_H_47_O_22_	903.8219	901		449; 323; 269	269	225	*Solanum tuberosum* [115]
**172**	Tannin	HHDP-hexose	C_20_H_18_O_14_	482.3485		483	465; 433; 387; 337; 153	135		*Myrtle* [30]; *Carpinus betulus* [63]
**173**	Gallotannin	Dehydro-galloyl-HHDP-hexoside *	C_28_H_24_O_16_	616.4806		617	303	257; 165	229; 158	*Terminalia arjuna* [116]
**174**	Ellagitannin	Pedunculagin [*bis*-HHDP [hexahydroxydiphenoyl]-O-hexoside; bis-HHDP hexose] *	C_34_H_24_O_22_	784.5412		785	599; 504; 284	501; 395; 319; 263		*Carpinus betulus* [63]; *Myrtle* [30]
**175**	Gallotannin	Di-O-galloyl-HHDP-glucose [Heterophylliin A; Pedunculagin II]	C_34_H_26_O_22_	786.5570	785		483; 767; 633; 418; 301	313; 211		*Juglans regia* [79]; *Punica granatum* [86]; *Eucalyptus* [102]; *Rosa rugosa* [117]
**176**	Gallotannin	Di-galloyl-HHDP-hexose *	C_34_H_26_O_22_	786.5570	785		483; 767; 633; 418; 301	313; 211		*Carpinus betulus* [63]
**177**	Trans-cinnamic acid	Cinnamic acid (Trans-cinnamic acid; Phenylacrylic acid)	C_9_H_8_O_2_	148.1586		149	119			Honey [83]; Potato [116]; *Lonicera caerulea* [55]; *Ribes triste* [73]
**178**	Hydroxybenzoic acid (Phenolic acid)	Protocatechuic acid	C_7_H_6_O_4_	154.1201		155	135			*Inula gaveolens* [25]; *Eucalyptus* [102]; *Ribes meyeri* [27]; *Vaccinium macrocarpon* [29]; *Ribes pauciflorum* [73]; *Camellia kucha* [71]; *Lepechinia* [63]; *Rosa rugosa* [52]; *Ocimum* [59]
**179**	Hydroxybenzoic acid (Phenolic acid)	Gallic acid	C_7_H_6_O_5_	170.1195		171	139			*Carpinus betulus* [63]; *Inula gaveolens* [25]; *Ocimum* [59]; *Juglans mandshurica* [62]; *Eucalyptus* [102]; *Vaccinium macrocarpon* [29]; *Andean blueberry* [28]; *Ribes meyeri* [27]; *Camellia kucha* [71]; *Rosa rugosa* [52]; *Rosa rugosa* [91]
**180**	Phenolic acid	Ethyl protocatechuate [3,4-Dihydroxybenzoic Acid Ethyl Ester]	C_9_H_10_O_4_	182.1733		183	153; 123	125		*Ocimum* [59]; *Medicago varia* [17]
**181**	Phenolic acid	Veratric acid [3,4-Dimethoxybenzoic acid]	C_9_H_10_O_4_	182.1733		183	171; 153; 123	125; 134		*Rosa rugosa* [52]
**182**	Phenolic acid	3,5-Dimethoxybenzoic acid	C_9_H_10_O_4_	182.1733		183	171; 153; 123	125; 134		*Rosa rugosa* [52]
**183**	Methylbenzoic acid	Methylgallic acid [Methyl gallate] *	C_8_H_8_O_5_	184.1461		185	153; 127	135; 125		*Andean blueberry* [28]; *Lonicera caerulea* [55]; Grape [110]
**184**	Phenolic acid	2,3,4,5-Tetrahydroxybenzoic acid [2-Hydroxygallussaure; 3,4,5-Trihydroxysalicylic acid]	C_7_H_6_O_6_	186.1189		187	145; 163; 127	127		PubChem
**185**	Phenolic acid	Hydroxygallic acid *	C_7_H_8_O_6_	188.1348		188	170; 146	118; 143		*Embelia* [80]
**186**	Hydroxycinnamic acid	3,4-Dimethoxy cinnamic acid	C_11_H_12_O_4_	208.2106		209	177	145		*Embelia* [80]; *Rosa rugosa* [52]
**187**	Hydroxycinnamic acid	Hydroxyferulic acid	C_10_H_10_O_5_	210.1834		211	193; 175; 147	175; 147; 129	129	*Andean blueberry* [28]; *Rosa davurica* [81]; *Lonicera caerulea* [55]; *Ribes dikuscha* [73]
**188**	Phenolic acid	Ibuprofen *	C_13_H_18_O_2_	206.2808		207	161	143	129	*Juglans mandshurica* [62]
**189**	Hydroxycinnamic acid	Sinapic acid [Sinapinic acid; 3,5-Dimethoxy-4-hydroxycinnamic acid; trans-Sinapic acid]	C_11_H_12_O_5_	224.21		225	207; 189; 161	189; 175; 161	171; 143	Potato [115]; *Vaccinium macrocarpon* [29]; Cranberry [75]; *Andean blueberry* [28]; *Ribes triste* [73]; *Vitis vinifera* [85]; *Rosa rugosa* [52,77]
**190**	Hydroxybenzoic acid (Phenolic acid)	Ellagic acid [Benzoaric acid; Elagostasine; Lagistase; Eleagic acid] *	C_14_H_6_O_8_	302.1926	301		257	229	201	*Ribes meyeri* [27]; Strawberry [87]; Grape [110]
**191**	Phenolic acid	Coumaroyl coumaric acid	C_18_H_14_O_5_	310.3008		311	163; 293	145	127	*Andean blueberry* [28]
**192**	Hydroxycinnamic acid	p-Coumaric acid-O-hexoside [Trans-p-Coumaric acid 4-glucoside]	C_15_H_18_O_8_	326.2986		327	309; 291; 276; 251; 230	261; 241; 175	227	*Carpinus betulus* [63]; *Inula viscosa* [54]; *Ribes meyeri* [27]; Cranberry [78]; *Ribes magellanicum* [65]; *Vaccinium macrocarpon* [72]; *Andean blueberry* [28]; *G. linguiforme* [61]; Grape [110]
**193**		Caffeic acid derivative	C_16_H_18_O_9_Na	377.2985	377		341; 281; 215	179; 161; 119	143	*Embelia* [80]
**194**	Phenolic acid	Caffeoylquinic acid derivative	C_17_H_18_O_10_	382.3188		383	207; 249	179; 123	150; 123	PubChem
**195**	Hydroxycinnamic acid	Sinapic acid-O-hexoside	C_17_H_22_O_10_	386.3576		387	369; 207; 161	189; 149	159	*Andean blueberry* [28]; *Ribes magellanicum* [65]; Cranberry [78]
**196**	Hydroxycinnamic acid	1-O-Sinapoyl-beta-D-glucose	C_17_H_22_O_10_	386.3576		387	369; 207; 161	189; 149	159	*Vitis vinifera* [85]; Cranberry [75]
**197**	Hydroxycinnamic acid	Hydroxycinnamic acid derivative	C_18_H_24_O_12_	432.3760	431		385; 223; 161	205; 153	187	PubChem
**198**	Phenolic acid	Ellagic acid pentoside [Ellagic acid 4-*O*-xylopyranoside]	C_19_H_14_O_12_	434.3073		435	303	257; 165	229; 201	*Juglans regia* [79]; *Carpinus betulus* [63]; Strawberry [82]; Strawberry [87]; *Ribes pauciflorum* [73]
**199**	Phenolic acid	Ellagic acid-*O*-arabinoside	C_19_H_14_O_12_	434.3073	433		301	179	151	*Phyllanthus* [118]
**200**	Phenolic acid	Methyl ellagic acid-*O*-pentose	C_20_H_16_O_12_	448.3338		449	317	285	257	Pecan [119]; Strawberry [82]
**201**	Phenolic acid	Ellagic acid-*O*-deoxyhexoside	C_20_H_16_O_12_	448.3338		449	303; 173	257		Strawberry [87]; *Punica granatum* [86]
**202**	Phenolic acid	Ellagic acid-*O*-hexoside	C_20_H_16_O_13_	464.3332		465	303	257; 165	229; 201	*Carpinus betulus* [63]; *Myrtle* [30]; *Eucalyptus* [102]; Strawberry [82]; *Punica granatum* [86]; *Pelargonium endlicherianum* [98]
**203**	Phenolic acid	Ellagic acid-*O*-glucoside	C_20_H_16_O_13_	464.3332		465	303	257; 165	229; 201	*Punica granatum* [86]
**204**	Phenolic acid	Ellagic acid-*O*-glucuronide	C_22_H_22_O_12_	478.4029		479	303	257; 229	201	*Phyllanthus* [118]
**205**	Phenolic acid	Coumaroyl-methylgalloyl hexose	C_23_H_24_O_12_	492.4295	491		345; 183	183	167	*Carpinus betulus* [63]
**206**	Phenolic acid	Caffeoyl hexosyl trihydroxymethoxyphenyl propanoic acid	C_25_H_28_O_14_	552.4814	551		345; 183	183	168; 124	*Andean blueberry* [28]
**207**	Phenolic acid	Ellagic acid-galloyl-hexoside	C_28_H_24_O_16_	616.4806		617	303	303; 257; 165	229; 158	*Punica granatum* [86]
**208**	Phenolic acid	Ellagic acid-*O*-dihexoside	C_26_H_26_O_18_	626.4738		627	465; 303	257; 165	229	*Phyllanthus* [118]; *Carpinus betulus* [63]; *Punica granatum* [86]
**209**	Phenolic acid	5-O-p-coumaroyl-4-O-caffeoyl-4-methylpentanoic acid-5-hydroxy-3-quinate		694.25		695	353; 261	335; 261; 173; 123	243; 159	*Phoenix dactylifera* [120]
**210**	Dihydrochalcone	Phloretin [Dihydronaringenin; Phloretol]	C_15_H_14_O_5_	274.2687		275	256	229	159	*Eucalyptus* [102]; *G. linguiforme* [61]; *Rosa rugosa* [81]; *Lonicera caerulea* [55,120]
**211**	Dihydrochalcone	Phlorizin [Phloridzin; Phlorizoside; Floridzin: phlorrhizin; Phloretin 2′-Glucoside; Phloretin-O-hexoside] *	C_21_H_24_O_10_	436.4093	435		273	167; 123	123	Potato [115]; *Eucalyptus* [102]; *Andean blueberry* [28]; *A. cordifolia* [61]
**212**	Stilbene	3-Hydroxyresveratrol [Piceatannol]	C_14_H_12_O_4_	244.2427		245	218; 186; 145	158	141	*G. linguiforme* [61]; Grape [110]; *Rosa rugosa* [81]; *Ribes dikuscha* [73]
**213**	Coumarin	Umbelliferone hexoside *	C_15_H_16_O_8_	324.2827		325	271; 253; 223; 181	241; 181; 145	153	*G. linguiforme* [61]; *Lonicera caerulea* [55]
**214**	Hydroxycoumarin	Fraxidin [2H-1-Benzopyran-2-One, 8-Hydroxy-6,7-Dimethoxy-] *	C_11_H_10_O_5_	222.1941		224	207; 189	161; 143	143	*Rat plasma* [121]
**215**	Coumarin glucoside	Fraxin (Fraxetin-8-O-glucoside) *	C_16_H_18_O_10_	370.3081		371	352; 322; 213; 118	322; 292; 165	235; 168; 135	Potato [116]; *Actinidia deliciosa* [122]
**216**	Lignan	Pinoresinol	C_20_H_22_O_6_	358.3851	357		269; 225; 181	181		*Passiflora incarnata* [69]; *Rosa rugosa* [81]
**217**	Lignan	Syringaresinol	C_22_H_26_O_8_	418.4436		419	255	239	211	*Annona montana* [123]
	OTHERS									
**218**		2,3-Dihydro-3,5-dihydroxy-6-methyl-4(H)-pyran-4-one [DDMP]	C_6_H_8_O_4_	144.1253		145	129	112		*Radix polygoni multiflori* [99]
**219**	Aliphatic amino acid	L-Glutamic acid [L-Glutamate]	C_5_H_7_NO_4_	145.1134		145	127			*Medicago truncatula* [124]
**220**	Glutamine family amino acid	Glutamine	C_5_H_10_N_2_O_3_	146.1445		147	129	111		*Triticum aestivum* [125]
**221**	Benzaldehyde	Vanillin	C_8_H_8_O_3_	152.1473		153	133			Potato [115]
**222**	Amino acid	L-Histidine	C_6_H_9_N_3_O_2_	155.1546		157	129	111		*Triticum aestivum* [125]; *Medicago truncatula* [124]; *Camellia kucha* [71]; *Actinidia deliciosa* [122]; *Lonicera caerulea* [55]; *Lonicera caerulea* [119]; *Ribes dikuscha* [73]
**223**	Aromatic amino acid	Phenylalanine [L-Phenylalanine]	C_9_H_11_NO_2_	165.1891		166	120; 147	120		*Triticum aestivum* [125]; *Medicago truncatula* [124]; *Medicago varia* [17]; *Juglans mandshurica* [62]; *Passiflora incarnata* [69]; *G. linguiforme* [61]; *Camellia kucha* [71]
**224**	Amino acid	L-theanine [Theanine; Theanin; N-Ethyl-L-glutamine]	C_7_H_14_N_2_O_3_	174.1977		175	158	140		*Camellia kucha* [71]; *Medicago varia* [17]; *Ribes aureum* [73]
**225**		L-Ascorbic acid [Vitamin C]	C_6_H_8_O_6_	176.1241		177	145			*Phoenix dactylifera* [120]
**226**	Tricarboxylic acid	Citric acid [Anhydrous; Citrate]	C_6_H_8_O_7_	192.1235	191		111; 173			strawberry [82]; *Stevia rebaudiana* [84]
**227**	Polyhydroxycarboxylic acid	Quinic acid	C_7_H_12_O_6_	192.1666	191		111; 173	111		*Inula graveolens* [25]; *Artemisia* [23]; *Ribes meyeri* [27]; *Andean blueberry* [28]; *Eucalyptus* [102]; *C. edulis* [61]; pear [90]; *Vitis vinifera* [85]; *Camellia kucha* [71]
**228**	Alpha,omega dicarboxylic acid	Sebacic acid [Decanedioic acid] *	C_10_H_18_O_4_	202.2475		203	175	143	111	*Lonicera caerulea* [55]; *Ribes dikuscha* [73]
**229**	Essential amino acid	L-Tryptophan [Tryptophan; (S)-Tryptophan] *	C_11_H_12_N_2_O_2_	204.2252		205	145			*Triticum aestivum* [125]; *Medicago truncatula* [124]; Strawberry [82]; *Passiflora incarnata* [69]; *Camellia kucha* [71]
**230**	Polysaccharides	Glucaric acid [D-Glucaric acid; Saccharic acid; D-Glutarate] *	C_6_H_10_O_8_	210.1388		210	193; 175; 147	175; 147	147; 129	Soybean [126]
**231**	Polysaccharides	Galactaric acid [Mucic acid; Saccharolactic acid; Galactarate] *	C_6_H_10_O_8_	210.1388		210	193; 175; 147	175; 147	147; 129	Soybean [126]; *Stevia rebaudiana* [84]
**232**	Alpha,omega dicarboxylic acid	Undecanedioic acid	C_11_H_20_O_4_	216.2741		217	199; 187; 171; 156; 137; 117	188; 160; 135		*G. linguiforme* [61]; *Jatropha* [53]
**233**	Sesquiterpenoid	Aristolone	C_15_H_22_O	218.3346		219	189	174	146	*Rumex hastatus* [127]
**234**	Sesquiterpenoid	Caryophyllene oxide [Caryophyllene-alpha-oxide]	C_15_H_24_O	220.3505		221	219; 207; 189; 171; 161			*Rosa davurica* [81]
**235**	Alpha,omega dicarboxylic acid	3,4,5-Trimethoxyphenylacetic acid	C_11_H_14_O_5_	226.2259	225		178; 161; 143; 119	161; 143; 131		*Rosa rugosa* [52]
**236**	Carboxylic acid	Myristoleic acid [Cis-9-Tetradecanoic acid]	C_14_H_26_O_2_	226.3550		227	209; 168	166; 139		*F. glaucescens* [61]; *Maackia amurensis* [18]
**237**	Sesquiterpenoid	Atractylenolide I	C_15_H_18_O_2_	230.3022		231	203; 185; 159	157; 145		*Atractylodes macrocephalae rhizoma* [128]; Chinese herbal formula Jian-Pi-Yi-Shen pill [19]
**238**	Quaianolide sesquiterpene lactone	Dehydrocostus Lactone	C_15_H_18_O_2_	230.3022		231	186; 158	168; 146	141	*Rosa davurica* [81]
**239**	Sesquiterpenoid	Atractylenolide II [Asterolide; 2-Atractylenolide]	C_15_H_20_O_2_	232.3181		233	186	168; 142		*Codonopsis Radix* [128]; Chinese herbal formula Jian-Pi-Yi-Shen pill [19]
**240**	Anthraquinone	1,8-Dihydroxy-anthraquinone [Chrysazin]	C_14_H_8_O_4_	240.2109		241	213	185	166	*Juglans mandshurica* [62]; *Ribes pauciflorum* [73]
**241**	Lactone	1beta-Hydroxyalantolactone	C_15_H_20_O_3_	248.3175		249	231; 189; 149	185; 129	158; 128	*Inula viscosa* [54]
**242**	Naphthoquinone	1,3,6,8-Tetrahydroxy-9(10H)-anthracenone	C_14_H_10_O_5_	258.2262		259	240; 213; 198; 184	191; 167; 139		*Juglans mandshurica* [62]; *Ribes pauciflorum* [73]
**243**	Plant hormone	Abscisic acid [Dormin; Abscisin II; (S)-(+)-Abscisic acid]	C_15_H_20_O_4_	264.3169		265	204	174	146; 112	Honey [83]
**244**	Aporphine alkaloid	Anonaine	C_17_H_15_NO_2_	265.3065		266	249	203	157	*Rosa rugosa* [81]; *Dracocephalum jacutense* [16]; *Lonicera caerulea* [55]
**45**	Ribonucleoside composite of adenine (purine)	Adenosine	C_10_H_13_N_5_O_4_	267.2413		268	250; 232	232; 186	186; 168	*Medicago varia* [17]
**246**	Omega-3-fatty acid	Stearidonic acid [6,9,12,15-Octadecatetraenoic acid; Moroctic acid]	C_18_H_28_O_2_	276.4137		278	261; 173; 115	215; 115	129	*G. linguiforme* [61]; *Jatropha* [53]
**247**	Omega-3-fatty acid	Linolenic acid (Alpha-Linolenic acid; Linolenate)	C_18_H_30_O_2_	278.4296		279	261	219	163	*Jatropha* [53]; *Maackia amurensis* [18]
**248**		Linoleic acid amide	C_18_H_33_NO	279.4607		280	262; 205; 149	149		PubChem
**249**	Terpenoid	Rugosic acid A	C_15_H_22_O_5_	282.3322		283	215; 153			*Rosa rugosa* [129]
**250**	Mixture of diastereomers	Fructose-leucine	C_12_H_23_NO_7_	293.3135		294	276; 235; 179	175; 189; 147	147	*Lonicera caerulea* [55]
**251**	Pterocarpan	3-Hydroxy-9,10-dimethoxypterocarpan	C_17_H_16_O_5_	300.3059		301	283; 259; 195	268; 240; 171	240	*Radix astragali* [56]; Chinese herbal formula Jian-Pi-Yi-Shen pill [19]
**252**	Aminoalkylindole	Tryptamine *	C_10_H_12_N_2_	160.2157		161	143			*Hylocereus polyrhizus* [130]; *Ribes triste* [73]
**253**	Unsaturated essential fatty acid	Oxo-eicosatetraenoic acid *	C_20_H_30_O_3_	318.4504		319	300	281; 149	249; 151	*F. potsii* [61]; *Ribes pauciflorum* [73]
**254**	Cyclohexenecarboxylic acid	Coumaroyl shiikimic acid	C_16_H_16_O_7_	320.2940		319	301; 173	127	142	*Andean blueberry* [28]; *Lonicera caerulea* [55]
**255**	Oxylipin	9,10-Dihydroxy-8-oxooctadec-12-enoic acid [oxo-DHODE; oxo-Dihydroxy-octadecenoic acid] *	C_18_H_32_O_5_	328.4437	327		229; 291; 171	211; 155	209; 183	*Lonicera caerulea* [55]; *Dracocephalum jacutense* [16]
**256**	Oxylipin	Trihydroxyoctadecadienoic acid *	C_18_H_32_O_5_	328.4437	327		229; 291; 211; 171	211; 155; 125	209; 183; 125	*Artemisia absinthium* [23]
**257**	Oxylipin	Trihydroxyoctadecenoic acid *	C_18_H_32_O_5_	328.4437	327		229; 291; 211; 171	211; 155; 125	209; 183; 125	*Artemisia absinthium* [23]
**258**	Oxylipin	13-Trihydroxy-Octadecenoic acid [THODE] *	C_18_H_34_O_5_	330.4596	329		229; 311; 171	211; 125	209; 183	*Jatropha* [53]; *Phoenix dactylifera* [120]
**259**		Dihydroxy eicosatrienoic acid	C_20_H_34_O_4_	338.4816		339	321; 303	275; 247; 235	207	*G. linguiforme*; *A. cordifolia*; *C. edulis* [61]
**260**	Xanthone	Paxanthone	C_19_H_16_O_6_	340.3267	339		324	309	291; 163	*Inula viscosa* [54]
**261**	Dammarane glycoside	Blumenol C-glucoside [Byzantionoside B; 9-Epi-Blumenol *C*-glucoside]	C_19_H_32_O_7_	372.4532		373	211; 193	193	175	*Passiflora incarnata* [69]
**262**	Cyclopentapyran; Iridoid	Loganic acid *	C_16_H_24_O_10_	376.3558		377	346; 277	246	245; 166	PubChem
**263**	Disaccharide	Trehalose (+FA adduct) CH2O2 (46.0254)	C_13_H_24_O_13_	388.3219	387		341	179		PubChem
**264**	Phytosterol	Ergosterol [Provitamin D2; Ergosterin]	C_28_H_44_O	396.6484		397	379; 273	213		*F. glaucescens* [61]; *Lonicera caerulea* [55]
**265**	Sterol	Beta-Sitosterin [Beta-Sitosterol]	C_29_H_50_O	414.7067	413		381; 301; 263	301; 263; 235	283; 245; 214	*F. herrerae* [61]; *Atractylodes macrocephalae Rhizoma* [128]; *Carduus pycnocephalus* [131]
**266**	Pentacyclic triterpene	Lupeol [Fagarasterol; Clerodol; Monogynol B; Lupenol] *	C_30_H_50_O	426.7174		427	409; 347; 251	347; 154	251	*Juglans mandshurica* [62]; *Carduus pycnocephalus* [131]
**267**	Sterol	Oxo-hydroxy sitosterol	C_29_H_48_O_3_	444.6896		445	427	335; 289; 261; 207	289; 261; 207; 133	*C. edulis*; *F. pottsii* [61]
**268**	Anabolic steroid	Vebonol *	C_30_H_44_O_3_	452.6686		453	435; 210	226; 336	210	*Hylosereus polyrhizus* [130]
**269**	Terpenoid	1-Hydroxy-3-oxours-12-en-28-oic acid	C_30_H_46_O_4_	470.6838		471	407; 201	205	176	Pear [90]
**270**	Terpenoid	Triterpenoid	C_30_H_46_O_4_	470.6838		471	407; 205	205	176	*Brazilian propolis* [132]
**271**	Ursane triterpene	Annurcoic acid *	C_30_H_46_O_5_	486.6832	485		467; 370	423	393	*Annurca apple* [133]; Pear [90]
**272**	Terpenes	Triterpenoid	C_30_H_48_O_5_	488.6991		489	453; 407; 353; 262	407; 335; 249	389; 335; 267; 201	PubChem
**273**	Triterpenoid	Euscaphic acid *	C_30_H_48_O_5_	488.6991	487		469; 425	439		Pear [90]
**274**	Triterpenoid	Tormentic acid [Jacarandic acid; Tomentic acid] *	C_30_H_48_O_5_	488.6991	487		469; 425	439		Pear [90]
**275**	Trihydroxy oleanolic acid	Arjungenin [2alpha, 19 alpha, 23-Trihydroxyoleanolic acid] *	C_30_H_48_O_6_	504.6985	503		485	410; 307; 191		PubChem
**276**	Trihydroxy oleanolic acid	Methyl arjunolate *	C_31_H_50_O_5_	502.7257		503	449; 259; 187	403; 335; 257	385; 321; 273	*G. linguiforme*; *C. edulis* [61]
**277**	Terpenoid	Triterpenoid		505		506	471; 407; 355; 320	407; 351; 247	201; 133	PubChem
**278**	Indole sesquiterpene alkaloid	Sespendole *	C_33_H_45_NO_4_	519.7147		520	184	125		*Hylosereus polyrhizus* [130]
**279**		*N*′,*N*′,*N*‴-Tri-*p*-coumaroyl spermidine	C_34_H_37_N_3_O_6_	583.6741		584	339; 307; 216; 175	307; 275; 192	279	*Rosa rugosa* [1]
**280**		*N*′,*N*′,*N*‴-Di-*p*-*c*oumaroyl caffeoyl spermidine	C_34_H_37_N_3_O_7_	599.6735	598		503	485; 389		*Rosa rugosa* [1]
**281**		Pheophorbide a	C_35_H_34_N_4_O_6_	606.6677		607	547; 461	461; 433	433	[134]
**282**	Terpenoid	Triterpenoid		667.234		668	453; 425; 351; 279	407; 323; 243; 201	389; 335; 277; 227	PubChem
**283**	Iridoid	p-Coumaroyl monotropein hexoside		698.8810		699	537; 365	375; 331; 259; 203		*Vaccinium myrtillus* [135]

* Compounds identified for the first time in *R. acicularis*, *R. amblyotis*, and *R. rugosa*.
